# Hypofractionated stereotactic boost in intermediate risk prostate carcinoma: Preliminary results of a multicenter phase II trial (CKNO-PRO)

**DOI:** 10.1371/journal.pone.0187794

**Published:** 2017-11-30

**Authors:** David Pasquier, Philippe Nickers, Didier Peiffert, Philippe Maingon, Pascal Pommier, Thomas Lacornerie, Geoffrey Martinage, Emmanuelle Tresch, Eric Lartigau

**Affiliations:** 1 Centre Oscar Lambret, Academic Department of Radiation Oncology, University Lille II, Lille, France; 2 CRISTAL UMR CNRS 9189, Université Lille1, M3, Avenue Carl Gauss, Villeneuve-d'Ascq, France; 3 Institut de Cancérologie de Lorraine-Alexis Vautrin, Nancy, France; 4 La Pitié Salpêtrière Charles Foix, UPMC, Paris, France; 5 Centre Leon Berard, Department of Radiation Oncology, Lyon, France; 6 Department of biostatistics, Centre Oscar Lambret, Lille, France; German Cancer Research Center (DKFZ), GERMANY

## Abstract

**Purpose:**

Dose escalation may improve curability in intermediate-risk prostate carcinoma. A multicenter national program was developed to assess toxicity and tumor response with hypofractionated stereotactic boost after conventional radiotherapy in intermediate-risk prostate cancer.

**Methods and material:**

Between August 2010 and April 2013, 76 patients with intermediated-risk prostate carcinoma were included in the study. A first course delivered 46 Gy by IMRT (68.4% of patients) or 3D conformal radiotherapy (31.6% of patients). The second course delivered a boost of 18 Gy (3x6Gy) within 10 days. Gastrointestinal (GI) and genitourinary (GU) toxicities were evaluated as defined by NCI-CTCAE (v4.0). Secondary outcome measures were local control, overall and metastasis-free survival, PSA kinetics, and patient functional status (urinary and sexual) according to the IIEF5 and IPSS questionnaires.

**Results:**

The overall treatment time was 45 days (median, range 40–55). Median follow-up was 26.4 months (range, 13.6–29.9 months). Seventy-seven per cent (n = 58) of patients presented a Gleason score of 7. At 24 months, biological-free survival was 98.7% (95% CI, 92.8–99.9%) and median PSA 0.46 ng/mL (range, 0.06–6.20 ng/mL). Grade ≥2 acute GI and GU toxicities were 13.2% and 23.7%, respectively. Grade ≥2 late GI and GU toxicities were observed in 6.6% and 2.6% of patients, respectively. No grade 4 toxicity was observed.

**Conclusions:**

Hypofractionated stereotactic boost is effective and safely delivered for intermediate-risk prostate carcinoma after conventional radiation. Mild-term relapse-free survival and tolerance results are promising, and further follow-up is warranted to confirm the results at long term.

**Trial registration:**

**ClinicalTrials.gov** NCT01596816.

## Introduction

Prostate cancer remains the most common cancer among men in developed countries [1]. According to guidelines, treatment with curative intent must be proposed to patients with intermediate-risk prostate cancer and a life expectancy superior to ten years [2–3]. Radiotherapy plays a key role in the treatment of localized prostate cancer. Several randomized studies have shown that dose escalation significantly improves biochemical relapse free survival [3]. Intensity-modulated radiation therapy (IMRT) or brachytherapy as a boost are available for dose escalation. For instance, recent studies have reported 5-year biochemical control rates of 89–93% for intermediate-risk prostate cancer patients with high dose rate brachytherapy (HDR-BT) boost [4]. However, it is an invasive procedure with potential risk of adverse events for patients. Hypofractionated stereotactic boost (robotic- or linac- based) may be used as a non-invasive approach. Stereotactic body radiation therapy (SBRT) delivers high dose in a limited number of fractions while targeting volumes with high accuracy, in accordance with low α/β ratio prostate cancer confirmed by large recent clinical trials [5]. Despite higher doses per fraction, in patients treated exclusively by SBRT, the toxicity profile has been shown to be comparable to that with conventionally fractionated radiotherapy [6–7]. Additionally, the reduced planning target volume (PTV) allows optimally preservation of adjacent organs at risk. The aim of this multicenter phase II trial was to assess the tolerance of hypofractionated stereotactic boost radiation after normofractionated external beam radiation therapy (EBRT). In addition, patient functional status with a specific focus on functional outcomes including urinary and sexual symptoms, were evaluated.

## Materials and methods

### Patients

The study was approved by the local ethics board and all patients provided written consent to participate (ethic board: Comite de Protection des Personnes Nord Ouest IV; meeting May 11th 2010; reference CPP 10/24). Between August 2010 and April 2013, 76 patients from four centers (Centre Oscar Lambret, Lille; Centre Alexis Vautrin, Vandoeuvre les nancy; Centre Georges-François Leclerc, Dijon; Centre Leon Berard, Lyon) were prospectively included in this study. Inclusion criteria were histologically proven prostate adenocarcinoma and intermediate-risk prostate cancer according to the D’Amico classification (T2b and/or prostate specific antigen [PSA] between 10 et 20 ng/mL and/or a Gleason score of 7), ECOG performance status ≤1, prostatic volume ≤ 80 cc, no adenopathy (lymph node <1.5 cm on scanner or MRI and/or in lymph node dissection), no metastasis (bone scan), no prior prostate cancer treatment (prostatectomy, chemotherapy, hormonotherapy > 3 months) or pelvic irradiation, IPSS score ≤10, and a life expectancy ≥10 years. Exclusion criteria were absence of histological evidence, unfavorable stage (T2c and/or PSA >20 ng/mL and/or Gleason > 7), favorable stage (T1c T2a and PSA <10 ng/mL and Gleason <7), T3 and T4, prior history of uncontrolled disease and/or cancer treated within the last five years (except for basal cell skin carcinoma), contraindication to MRI, recurrent or metastatic disease, known allergy to gold, participation in another therapeutic trial assessing an experimental drug, and inability to undergo medical follow-up of the study for geographical, social or psychological reasons. The study was registered on clinicaltrial.gov after its initiation due to an administrative delay. All related CTs are registered.

### Treatment

The first clinical target volume (CTV1) was the prostate and proximal half of the seminal vesicles; CTV2 boost was prostate only. MRI and CT fusion based on fiducials was mandatory to define CTVs. Planning target volume for the first part of the treatment (PTV1) was defined as the addition of a 1 cm margin around the corresponding CTV1 and lowered to 0.5 cm posteriorly to spare the rectum. PTV2 was obtained by expanding 5 mm around CTV2. During the first part of the treatment, 23 fractions (2 Gy/session) were delivered over 42 days maximum, for a total dose of 46 Gy using 3D conformal radiotherapy or IMRT. The choice of radiation technique varied according to the center. During the second treatment part, hypofractionated stereotactic boost (3 fractions of 6 Gy) was delivered over 5 to 9 days (with at least 48 hours between sessions) for a total dose of 18 Gy. 95% of PTV received 18 Gy with a maximum of 21.2 Gy inside the GTV. Fiducials were implanted before the first radiation to allow daily image-guided radiation therapy (IGRT) (± tracking for patients treated with Cyberknife) during the two parts of the treatment.

#### Functional assessment

**Fig 1:**
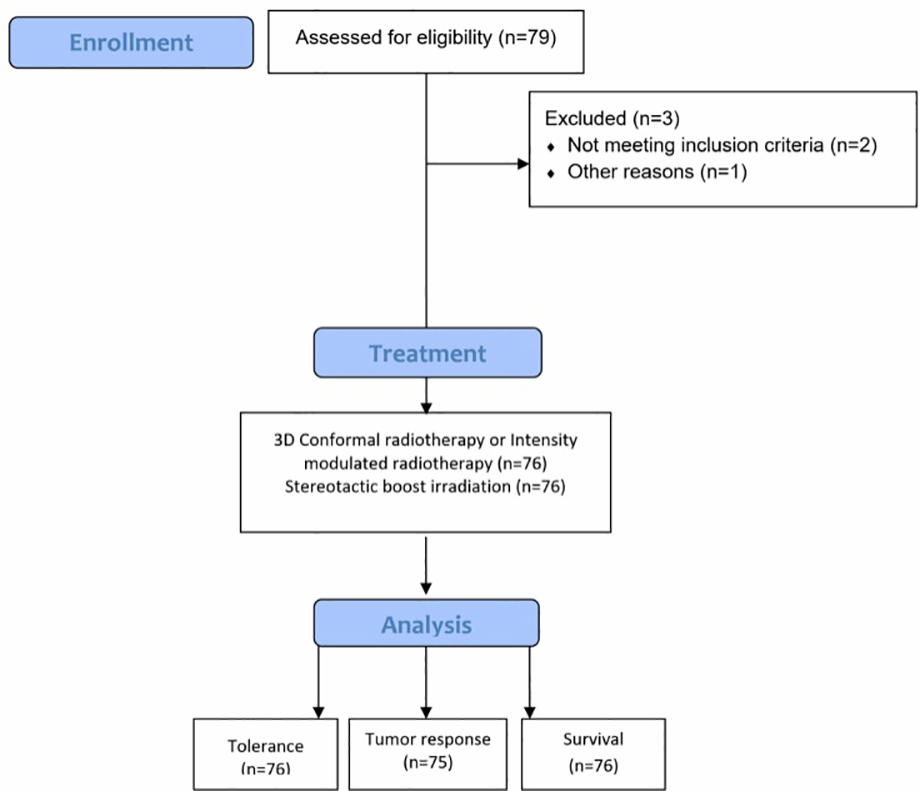
Flowchart diagram of the study.

The prevalence of erectile and urinary dysfunctions at inclusion, and at 3, 6, 9, 12, 18 and 24 months was determined in all patients by using the 5-item version of the International Index of Erectile Function (IIEF-5) [8] and the International Prostate Symptom Score (IPSS). The possible scores for the IIEF-5 range from 1 to 25 and a score >21 is considered as normal erectile function and ≤21, erectile dysfunction. According to this scale, erectile dysfunction was classified into five categories: non-interpretable (1–4), severe (5–10), moderate (11–15), mild (16–20), and normal erectile function (21–25). The total IPSS score can range from 0 to 35 and is classified as mild (0–7), moderate (8–19) or severe (20–35). IPSS and IIEF-5 proportions at baseline and 24 months were compared with each other using the McNemar test for paired data in the population of patients evaluable at baseline and at 24 months (Flowchart diagram in Fig 1). Questionnaires were self-administered and completed in French.

#### Endpoints and statistics

A single-step Fleming [9] design was used to calculate the sample size in this phase II study. The rate of grade 3–4 toxicity-free patients below which the treatment does not warrant further investigations (p0) was estimated at 85%. The minimal rate of grade 3–4 toxicity-free patients required for the treatment to be deemed efficient and interesting (p1) was estimated at 95%. With a type I error set to 5%, 76 evaluable patients were required to be recruited to achieve 90% power. The treatment was deemed interesting for further research if at least 70 out of 76 evaluable patients were free of grade 3–4 toxicity.

Primary outcomes were rectal and urinary toxicity according to the NCI-CTCAE v4.0 scale. Change from baseline in rectal functions was assessed every 3 months following boost irradiation for 1 year and then every 6 months for 2 years and then every year. Toxicities occurring within 6 months after the end of radiotherapy were deemed acute and events that occur beyond this period were defined as late toxicities. Actuarial cumulative toxicity incidence was calculated by the Kaplan-Meier method. Secondary outcome measures were local control of prostate cancer defined as non-progressive PSA according to Phoenix criteria (PSA failure was defined as nadir +2 ng/mL), and no clinical progression. Overall and metastasis-free survival were calculated using the Kaplan-Meier method. Other factors examined were PSA kinetics, sexual toxicity according to the IIEF-5 questionnaire, and urinary discomfort according to the IPSS questionnaire. IPSS and IIEF-5 proportions at baseline and 24 months were compared with each other using the McNemar test for paired data, in the population of patients evaluable at baseline and 24 months.

Categorical variables are described in terms of numbers and proportions (with 95% confidence interval [CI]). Continuous variables are summarized using medians and ranges. Stata v11.2 (StataCorp. 2009. Stata Statistical Software: Release 11. College Station, TX: StataCorp LP) was used for the statistical analyses.

## Results

The clinical and pathological characteristics of patients are presented in Table 1. The median age at diagnosis was 71 years (range, 45–84). A total of 58 patients (76.3%) presented with a Gleason score 7 (3+4 pattern n = 40, 4+3 pattern n = 18) and 18 patients (23.7%) had a Gleason score of 6. Patients did not receive any cancer treatment prior to radiotherapy.

**Table 1:** Demographic, clinical and pathological characteristics of patients at baseline.

Caracteristics (n = 76)	n	%
**Age (median, range)**	71 years (45–84)	
BMI		
Underweight (<18.5)	26	34
Normal (18.5–25)	32	42.1
Overweight (25–30)	12	15.8
Obese (≥30)	4	5.3
Unknown	2	2.6
Diabetes	2	2.6
Smoking history		
Non smoker	40	52.6
Previous smoker	30	39.5
Active smoker	6	7.9
**T TNM**		
T1c	33	43.4
T2a	16	21.1
T2b	24	31.6
T2c	3	3.9
**N TNM**		
N0	75	98.7
Nx	1	1.3
**M TNM**		
M0	76	100
**Gleason Score**		
6	18	23.7
7	58	76.3
+ 4	40	52.6
+ 3	18	23.7
**Rectal examination**		
Normal	30	40.5
Tumoral	44	59.5
Missing	2	
**WHO performance status**		
0	67	88.2
1	9	11.8

Abbreviations: WHO: world health organization; BMI: body mass index (kg/m^2^)

For the first part of the treatment, 52 (68.4%) patients were treated with IMRT and 24 (31.6%) with 3D conformal radiotherapy. The median total dose at the International Commission on Radiation Unit reference point was 46 Gy (range, 46.0–48.5) and the median duration of radiotherapy treatment was 34 days (range, 31–40). For the second part of the treatment, 60 patients (78.9%) received SBRT boost using the cyberknife system and 16 patients (21.1%) were treated with a linear accelerator (Linac). The median gap between the two courses was 5 days (range, 1–17 days). The median duration of stereotactic treatment was 8 days (range, 4–12). All patients were administered 18 Gy in three fractions, apart from four patients (four fractions) due to interruption of one of the sessions for technical reasons. The overall treatment time (from start of first radiotherapy to end of boost) was 45 days (median, range 40–55). The cumulative duration of conventional fractionated radiotherapy and stereotactic boost, excluding the time interval between the two courses was 42 days (range, 37–48).

**Fig 2:**
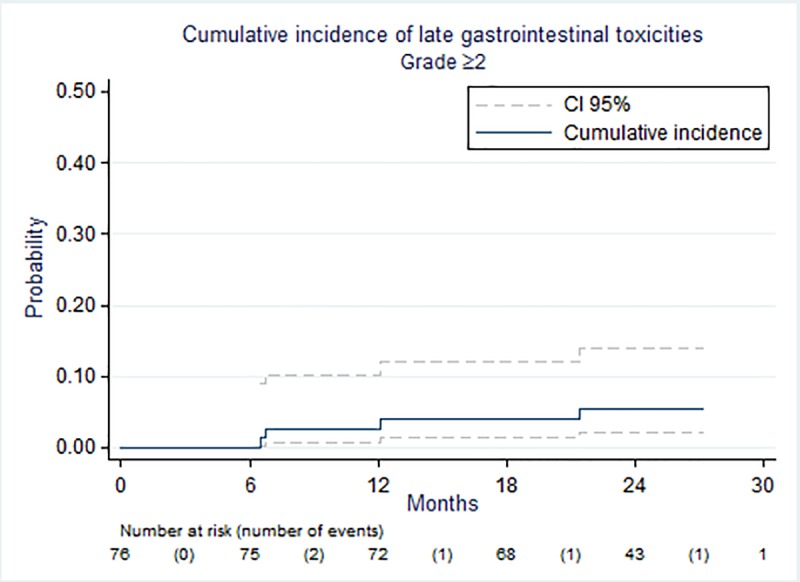
Actuarial cumulative incidence of grade ≥ 2 late gastrointestinal toxicities (CTCAE v4.0).

Median follow-up was 26.4 months (range, 13.6–29.9). Treatment-related acute or late toxicities are summarized in Table 2. Acute grade ≥2 GI toxicities were observed in 13.2% (n = 10) and dropped to 6.6% (n = 5) more than 6 months after radiotherapy. Most common acute and late GI toxicities included grade 2 diarrhea (n = 5, 6.6%) and grade 2 proctitis (n = 4, 5.3%). One patient developed treatment-related late grade 3 rectal bleeding. The cumulative incidence rates of late grade ≥2 GI complications at 12 and 24 months after the end of radiotherapy were 2.7% (95% CI, 0.7–10.2) and 5.5% (95% CI, 2.1–13.9), respectively (Fig 2), and zero for late grade ≥2 GU toxicity. Eighteen (23.7%) patients reported treatment-related acute grade ≥2 GU toxicities, two (2.6%) of which were grade 3, one dysuria and one nocturia. Most grade ≥2 toxicities resolved within 6 months and only one grade 2 micturition urgency and one grade 2 urinary incontinence were observed (Table 2).

**Table 2:** Treatment related acute and late toxicities (CTCAE v4.0).

	Acute toxicity (n = 76)			Late toxicity (n = 76)		
	Grade 2	Grade 3		Grade 2 Grade 3		
	n %	n	%	n	% n	%
Gastrointestinal disorders	10 13.2	0	0.0	4	5.3 1	1.3
Genitourinary disorders	16 21.1	2	2.6	2	2.6 0	0.0

No treatment-related acute or late grade 4 or grade 5 GI or GU toxicities were observed.

**Fig 3:**
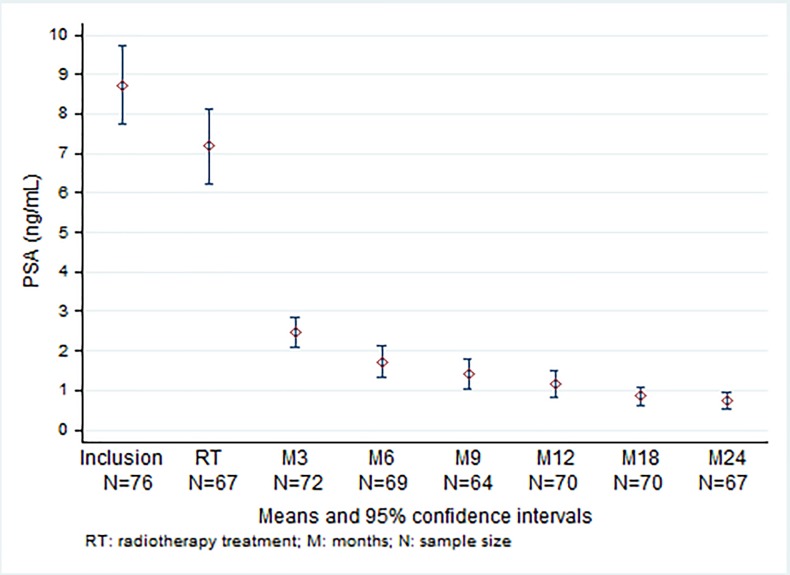
PSA at baseline, during radiotherapy treatment and during follow-up.

Median PSA at inclusion was 7.48 ng/mL (range, 2.91–22.40). Two patients presented with high serum PSA levels (> 20 ng/mL). PSA levels dropped to 6.21 ng/mL (range, 2.26–1.08 ng/mL) between the two courses and at 24 months, the levels further dropped to 0.46 ng/mL (range, 0.06–6.20) (Fig 3).

The decline rate of PSA was maximal in the first month (median -1.03 ng/mL/month), then gradually falling off with median values of -0.69, -0.48, -0.26 ng/mL/month for durations of 6 12, and 24 months following SBRT, respectively.

Overall survival and metastasis-free survival at 24 months were 97.4% (95% CI, 89.8–99.3) and 96.0% (95% CI, 88.2–98.7), respectively. At 24 months no local relapse was observed and biological free survival rate was 98.7% (95% CI, 90.7–99.8).

Median IPSS score at inclusion and at months 3, 6, 9, 12, 18 and 24 are presented in Table 3. Sixty patients responded for the IPSS QoL score both at inclusion and at 24 months. The score deteriorated from baseline for five patients, and two patients reported an improvement. QoL due to urinary symptoms showed no significant difference between baseline and 24 months (McNemar test, p = 0.26).

**Table 3:** Median IPSS and IIEF-5 scores at baseline and during follow-up.

	Baseline	M 3	M6	M9	M12	M18	M24
IPSS median score (range)	5 (0–22)	7 (0–20)	6 (0–19)	6 (0–20)	6 (0–21)	6 (0–23)	6 (0–23)
IIEF-5 median score (range)	15.5 (1–25)	7 (1–25)	12.5 (1–25)	5.5 (1–25)	9.5 (1–24)	4.5 (1–22)	9 (1–24)

Abbreviations: IPSS International Prostate Symptom Score; IIEF-5: International Index of Erectile Function; M: month.

Median IIEF-5 score at baseline and at months 3, 6, 9, 12, 18 and 24 are presented in Table 3. Of the 32 patients that reported normal to moderate dysfunction at baseline and responded to the questionnaire at 24 months, 8 patients (25%) reported a worsening of the symptoms; 24 (75%) patients remained in the same normal-to-moderate group. This difference was significant (p = 0.005) using a McNemar test for paired data.

## Discussion

A life expectancy greater than ten years is required to treat intermediate-risk prostate cancer patients with a curative intent according to the National Comprehensive Cancer Network and European Association of Urology guidelines [3]. A combined IMRT with short-term androgen deprivation therapy (ADT) can be administered in suitable patients [10–12].

However, for patients not suitable for ADT (due to comorbidities) or unwilling to accept ADT to preserve their sexual function, the recommended treatment is an IMRT at an escalated dose or a combination of IMRT and brachytherapy (BT). Indeed, several randomized studies have shown that dose escalation has a significant impact on survival without biochemical relapse, particularly in intermediate- and high-risk prostate cancer [3,13]. In Zelefsky et al’s retrospective series, high radiation dose levels were associated with improved biochemical tumor control and decreased risk of distant metastases [14–15]. Nevertheless, no randomized trials have shown that dose escalation results in an overall survival benefit.

While phase II trials with low dose rate BT (LDR-BT) boost have shown encouraging outcomes and toxicity profiles, high dose rate BT (HDR-BT) has been steadily growing in popularity as an alternative boost therapy. Further reports on toxicity or patient QoL with respect to HDR-BT boost are required to substantiate these results [16]. Two randomized trials compared external-beam radiation therapy (EBRT) + BT boost vs EBRT alone [17–18]. In the ASCENDE-RT trial, wherein patients received 12 months of ADT, the cumulative incidence of grade 3 GU late toxicity at 5 years was 18% in the LDR-BT arm compared with 5% for the EBRT only arm (P < 0.001). Urethral strictures requiring dilatation were responsible for half of the grade 3 GU toxicity events. The cumulative incidence of any pad usage at 5 years was 16.1% and 6.3% (p<0.001) in LDR-BT and EBRT only arms, respectively [17]. In the randomized trial by Hoskin et al. [18], the 7-year incidence rate for patients with any severe urinary symptom was similar in the HDR-BT boost and EBRT alone arms (p = 0.5). Over the first 8 years after treatment, the highest and lowest prevalence of severe urinary events was 14% and 4% for EBRT + HDRBT, and 10% and 0% for EBRT alone, respectively. The difference was significant only at 5.5 years (14% vs 0%, p = 0.02, respectively). In both trials, relapse-free survival was significantly higher in the BT boost arm. A matched pair analysis [4] showed that HDR-BT was associated with a significant reduction in biochemical failure (79.8% vs. 70.9% at 5 years, p = 0.0011) but with increased grade 3 urethral stricture (11.8% vs. 0.3%, p< 0.0001).

Orio et al. examined how the included men in the ASCENDE-RT trial were being treated in the United States from 2004 through 2012 [19]. Despite the evidence supporting its effectiveness, the use of EBRT + BT boost (compared to either EBRT or BT alone) from 2004 to 2012 declined from 15% to 8% in academic centers and from 19% to 11% in non-academic centers, respectively (p <0.0001 for both).

In view of these conflicting reports, SBRT could be proposed as a non-invasive alternative boost technique. SBRT allows high dose fractionation whilst optimally preserving organs at risk in accordance with low α/β ratio prostate cancer as confirmed by large recent clinical trial. A multicenter phase II trial on exclusive SBRT (35–36.25 Gy in 5 fractions) reported encouraging early results for the treatment of patients with localized prostate adenocarcinoma. A third of these patients presented with intermediate risk carcinoma [6]. Similarly in Katz et al’s exclusive SBRT series, 153 of the 515 patients presented with intermediate risk cancer. Patients were treated with a regimen of a five-fraction SBRT to a dose of 35–36.25 Gy. According to the authors, patients with unfavorable intermediate-risk disease (Gleason 4 + 3 = 7 or >1 intermediate-risk factors: cT2b, c, PSA 10–20, Gleason 3 + 4 = 7) had significantly worse outcomes after exclusive SBRT, and should be considered for clinical trials or treatment intensification [20].

To our knowledge, our series is the first reported prospective trial of dose escalation using SBRT as a boost for intermediate-risk prostate cancer patients. Despite the delivery of dose-escalated radiation, EBRT + SBRT boost was well tolerated by patients. Treatment-related acute GU and GI toxicity levels were low (Table 3) and most of them resolved within the two-year follow-up period. These results compare favorably with the toxicity profiles of other treatment approaches including BT boost or EBRT alone, but should be confirmed by a longer follow-up.

One other strength of our series is the absence of ADT to preserve potency and QoL and to avoid metabolic dysfunction. In a retrospective series of 108 patients (45 with an intermediate-risk cancer), the majority of them received ADT [21]. Patients were treated with SBRT (19.5 Gy in three fractions) followed by fiducial-guided IMRT (45–50.4 Gy). The 3-year actuarial biochemical control rates were 100% and 89.8% for intermediate- and high-risk patients, respectively. Erectile dysfunction was not assessed. At 2 years 13.7% and 5% of men complained from moderate to severe urinary and bowel dysfunctions respectively [21]. Another series reported results of SBRT as a boost after pelvic EBR in patients with high-risk prostate carcinoma. Ninety-seven patients were treated with either SBRT alone (n = 52) to a dose of 35–36.25 Gy in 5 fractions, or pelvic radiation to 45 Gy followed by SBRT boost of 19–21 Gy in 3 fractions (n = 45) [22]. Twenty-eight of these 45 patients received ADT. The 5-year biochemical disease-free survival for the combined high-risk group was 68%. The use of pelvic radiotherapy was associated with a significantly higher bowel toxicity (p = 0.001). Expanded prostate cancer index composite **(**EPIC) scores declined for the first six months and then returned to baseline values. In our series, biological relapse-free survival rate was 98.7% at two years. At 24 months, no local relapse was observed. Only one patient presented an early biochemical and metastatic recurrence one year after the end of radiotherapy without clinical local recurrence. The PSA level for this patient at inclusion was of 20 ng/mL with a doubling time <6 months before inclusion, suggesting aggressive disease at the inclusion. Using SBRT, the optimal schedule (SBRT alone or as a boost) for intermediate risk prostate cancer still remains unknown.

With higher rates of survival in prostate cancer patients, QoL has become an important outcome-measure for treatment decision-making. An increasing number of studies are focusing on measures reported by the patients rather than by the physicians (i.e. patient reported outcomes [PRO]). When this study was launched, PRO were rarely used and reported in phase II trials. The two questionnaires used in our series (IPSS and IIEF-5) focused on sexual and urinary functions, two domains that are most affected by radiotherapy. The two questionnaires were used throughout the follow-up to show a course of symptom development. Only 8% of the patients reported a worsening of the QoL due to urinary symptoms at 2 years. The impact of treatment-related side effects on erectile function was also encouraging. Of the 32 patients that reported normal to moderate dysfunction before treatment, 8 patients reported a worsening of the symptoms at 2 years. Nevertheless, the low number of patients does not allow drawing any definitive conclusions.

Despite these results, and due to the short follow-up, late toxicity and relapse-free survival are difficult to compare to other techniques. Twelve- and 24-month median PSA were 0.8 ng/mL (range, 0.13–10.00) and 0.46 ng/mL (range, 0.06–6.20), respectively. Recent studies have reported a relationship between post-radiotherapy nadir PSA nadir inferior to 1–2 ng/mL and long-term relapse-free survival [23–24]. In our series, PSA levels dropped significantly below this threshold value.

## Conclusion

Despite the limited follow-up, our results suggest that hypofractionated stereotactic boost radiation after normofractionated radiotherapy allows a high rate of local and biochemical control with low rate of GI and GU toxicity. Longer follow-up is necessary to confirm late toxicities and outcomes.

## Supporting information

S1 Trend Checklist (PDF)

S1 Consort Diagram (DOC)

S1 Protocol (DOC)
