# Supplementary material for: Hypofractionated stereotactic boost in intermediate risk prostate carcinoma: Preliminary results of a multicenter phase II trial (CKNO-PRO)
Source: PLoS One. 2017 Nov 30;12(11):e0187794. doi: 10.1371/journal.pone.0187794 (PMC5708754; doi:10.1371/journal.pone.0187794)
Supplement: S1 Consort Diagram — (DOC) [file pone.0187794.s002.doc]

**Flow Diagram**

**Analysis**

**Enrollment**

**Treatment**

Assessed for eligibility (n=76)

Excluded (n=3)

♦  Not meeting inclusion criteria (n=2)

♦  Other reasons (n=1)

Tolerance (n=76)

3D Conformal radiotherapy (n=76)

Stereotactic boost irradiation (n=75)

Tumor response (n=75)

Survival (n=76)
