# Supplementary material for: Hypofractionated stereotactic boost in intermediate risk prostate carcinoma: Preliminary results of a multicenter phase II trial (CKNO-PRO)
Source: PLoS One. 2017 Nov 30;12(11):e0187794. doi: 10.1371/journal.pone.0187794 (PMC5708754; doi:10.1371/journal.pone.0187794)
Supplement: S1 Protocol — (DOC) [file pone.0187794.s003.doc]

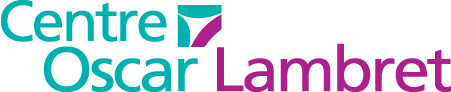


CLINICAL STUDY PROTOCOL

Protocol N°: 2009-01

**National Clinical Research Program 2009 (PHRC 2009)**

**Project n° 19-006**

**Protocol Title:**

**intermediate risk prostate cancer : phase II prospective studY evaluating hypofractionated stereotactic**

**boost irradiation**

**Study code:** CKNO-PRO-0901

**ClinicalTrials.gov identifier: NCT01596816**

| Sponsor: | **Centre Oscar Lambret**  3, rue Frédéric Combemale  BP 307  59020 LILLE CEDEX  **Tel:** (33)3 20 29 59 18 – **Fax**: (33)3 20 29 59 71 |
| --- | --- |

| **Principal Investigators:** | **Professor Eric LARTIGAU**  ***Centre Oscar Lambret & Université Lille II***  University Radiation Oncology Department  3 rue F. Combemale  BP 307  59020 LILLE CEDEX  Tel: (33)3 20 29 55 98 – Fax: (33)3 20 29 59 71  email: [e-lartigau@o-lambret.fr](mailto:e-lartigau@o-lambret.fr)  Professor Olivier CHAPET  ***Centre Hospitalier Lyon Sud***  Department of Radiation Oncology  69310 PIERRE BENITE  Tel: (33)3 78 86 42 60- Fax: (33)3 78 86 42 65  email: olivier.chapet@chu-lyon.fr |
| --- | --- |

**Confidentiality clause**

*The information contained in this document is the property of the Centre Oscar LAMBRET (COL). It has been provided to you confidentially to be examined by yourself, your team, the members of the concerned CPP and the Administrative authorities.*

*The information contained herein must not be communicated to third parties without prior written consent from the COL, with the exception of those elements required to obtain the informed consent of individuals who may participate in the research.*

Version n° 6 of January 5, 2009, approved by the CPP North-West IV on……….

and the AFSSAPS on………

# APPROVAL AND SIGNATORIES OF THE PROTOCOL

*Protocol N° CKNO-PRO-0901*

Protocol title: ***Prostate cancer, intermediate risk prognosis : Prospective phase II study evaluating hypofractionated stereotactic boost irradiation***

Date Signature

***Professor Eric LARTIGAU***  **/______________/ /______________________/**

###### Principal Investigator

***Professor Olivier CHAPET***  **/______________/ /______________________/**

###### Principal Investigator

***Yvette VENDEL* /______________/ /______________________/**

*CRA Sponsor*

***Charles Fournier* /______________/ /______________________/**

###### Biostatistician

***Stéphanie Clisant*** **/______________/ /______________________/**

*Manager of the Clinical Research Integrated Unit*

***Doctor Bernard Leclercq*** **/______________/ /______________________/**

*Director*

**PROTOCOL APPROVAL**

I acknowledge that I am aware of the entirety of protocol N° ***CKNO-PRO-0901*** (N° protocol: **2009-01)** and I am committed to conducting this protocol in conformance with Good Clinical Practices, the Public Health Law of August 9, 2004 and the application decree of April 26, 2006.

I assume the responsibilities incumbent on me as Principal Investigator, namely:

- The collection of informed consent, dated and signed by the patients before any selection procedure in the protocol,
- the validation of case report forms, completed for each of the patients included in the study,
- direct access to the source documents for verifications done by the monitor mandated by the sponsor,
- the archiving of essential study documents for a minimum period of 15 years.

| **Establishment** | **Principal Investigator's name** | **Date and signature** |
| --- | --- | --- |
|  |  |  |

**TABLE OF CONTENTS**

*1 APPROVAL AND SIGNATORIES OF THE PROTOCOL* [*2*](#__RefHeading___Toc219020369)

2 LIST OF PARTICIPATING CENTERS [5](#__RefHeading___Toc219020370)

3 SYNOPSIS [7](#__RefHeading___Toc219020371)

4 INTRODUCTION AND STUDY RATIONALE [10](#__RefHeading___Toc219020372)

4.1 Incidence [10](#__RefHeading___Toc219020373)

4.2 Mortality [10](#__RefHeading___Toc219020374)

4.3 Study Justification [10](#__RefHeading___Toc219020375)

4.3.1 Radiotherapy for localized prostate cancer [10](#__RefHeading___Toc219020376)

4.3.2 Hypofractionated Radiation Therapy [11](#__RefHeading___Toc219020377)

4.3.3 Importance of the irradiation total dose [11](#__RefHeading___Toc219020378)

4.3.4 Stereotactic radiation therapy concept [12](#__RefHeading___Toc219020379)

4.3.5 Stereotactic radiotherapy of prostate cancer [12](#__RefHeading___Toc219020380)

5 STUDY OBJECTIVES [13](#__RefHeading___Toc219020381)

5.1 Primary objective [13](#__RefHeading___Toc219020382)

5.2 Secondary objectives [13](#__RefHeading___Toc219020383)

6 STUDY DESIGN [14](#__RefHeading___Toc219020384)

6.1 Methodology [14](#__RefHeading___Toc219020385)

6.2 Inclusion Criteria [14](#__RefHeading___Toc219020386)

6.3 Exclusion criteria [14](#__RefHeading___Toc219020387)

6.4 Early study withdrawal criteria [15](#__RefHeading___Toc219020388)

6.5 Termination study criteria [15](#__RefHeading___Toc219020389)

6.6 Assessment visit [15](#__RefHeading___Toc219020390)

6.6.1 Inclusion examination [15](#__RefHeading___Toc219020391)

6.6.2 Evaluation during treatment [16](#__RefHeading___Toc219020392)

6.6.3 Post-treatment visit [16](#__RefHeading___Toc219020393)

6.6.4 Follow-up visit [16](#__RefHeading___Toc219020394)

6.6.5 End of study evaluation [17](#__RefHeading___Toc219020395)

6.6.6 Long term evaluation [17](#__RefHeading___Toc219020396)

6.7 Assessment Criteria [17](#__RefHeading___Toc219020397)

6.7.1 Tolerance evaluation [17](#__RefHeading___Toc219020398)

6.7.2 Evaluation of tumor efficacy [19](#__RefHeading___Toc219020399)

6.7.3 Evaluation of biological response [20](#__RefHeading___Toc219020400)

6.7.4 Evaluation of technical criteria [20](#__RefHeading___Toc219020401)

6.7.5 Evaluation of PSA kinetics during treatment [20](#__RefHeading___Toc219020402)

6.7.6 Evaluation of sexual dysfunction [20](#__RefHeading___Toc219020403)

6.7.7 Evaluation of urinary discomfort [21](#__RefHeading___Toc219020404)

6.8 Statistical analysis and determination of the number of patients [21](#__RefHeading___Toc219020405)

6.8.1 Data management [21](#__RefHeading___Toc219020406)

6.8.2 Statistical analysis [21](#__RefHeading___Toc219020407)

6.8.3 Determination of the number of subjects [21](#__RefHeading___Toc219020408)

6.8.4 Criteria for termination of the study [22](#__RefHeading___Toc219020409)

7 TREATMENT [22](#__RefHeading___Toc219020410)

7.1 Patient enrollment [22](#__RefHeading___Toc219020411)

7.2 FIRST PHASE : Conformal 3 D radiotherapy with or without intensity modulation [22](#__RefHeading___Toc219020412)

7.2.1 Patient positioning [22](#__RefHeading___Toc219020413)

7.2.2 Preparation for treatment – Placement of intra-prostatic markers (fiducials) [23](#__RefHeading___Toc219020414)

7.2.3 Images acquisition [23](#__RefHeading___Toc219020415)

7.2.4 Identification of target volumes [23](#__RefHeading___Toc219020416)

7.2.5 Localization of the prostate [23](#__RefHeading___Toc219020417)

7.2.6 Dosimetry [24](#__RefHeading___Toc219020418)

7.2.7 Doses to critical organs [24](#__RefHeading___Toc219020419)

7.3 SECOND PHASE : Hypofractionated stereotactic boost irradiation [25](#__RefHeading___Toc219020420)

7.3.1. By linear accelerator [25](#__RefHeading___Toc219020421)

7.3.2 By CyberKnife [26](#__RefHeading___Toc219020422)

7.4 Independent Data Management Comittee (IDMC) [28](#__RefHeading___Toc219020423)

7.5 Subsequent antitumor treatments [28](#__RefHeading___Toc219020424)

7.6 Concomitant treatments [28](#__RefHeading___Toc219020425)

8 REGULATORY AND ETHICAL ASPECTS [28](#__RefHeading___Toc219020426)

8.1 Study management and sponsor's responsibilities (COL) [28](#__RefHeading___Toc219020427)

8.2 Study management and investigator's responsibilities [28](#__RefHeading___Toc219020428)

8.3 Committee for the Protection of Persons (CPP) [29](#__RefHeading___Toc219020429)

8.4 Participant information and consent [29](#__RefHeading___Toc219020430)

8.5 Independent monitoring committee [29](#__RefHeading___Toc219020431)

8.6 Confidentiality [29](#__RefHeading___Toc219020432)

9 OPERATIONAL MANAGEMENT OF THE STUDY [30](#__RefHeading___Toc219020433)

9.1 Study organization [30](#__RefHeading___Toc219020434)

9.2 Research costs and additional costs [30](#__RefHeading___Toc219020435)

9.3 Data collection and input - Study follow-up - Monitoring [30](#__RefHeading___Toc219020436)

9.4 Quality Assurance [30](#__RefHeading___Toc219020437)

9.5 Data ownership and publication [30](#__RefHeading___Toc219020438)

10 BIBLIOGRAPHY [31](#__RefHeading___Toc219020439)

11 APPENDICES [34](#__RefHeading___Toc219020440)

Appendix 1: Study outline [35](#__RefHeading___Toc219020441)

Appendix 2: Study timelines [36](#__RefHeading___Toc219020442)

Appendix 3: Living conditions [37](#__RefHeading___Toc219020443)

Appendix 4: TNM classification regarding prostate cancer [38](#__RefHeading___Toc219020444)

Appendix 5: Gleason Score [39](#__RefHeading___Toc219020445)

Appendix 6: Recommendations for planning CT-Scan before treatment [40](#__RefHeading___Toc219020446)

Appendix 7: marker specifications and placement recommendations for tracking [41](#__RefHeading___Toc219020447)

Appendix 8: Informational letter to patient and consent form [43](#__RefHeading___Toc219020448)

Appendix 9: Declaration forms for serious adverse events [47](#__RefHeading___Toc219020449)

Appendix 10 : NCI-CTCAE Version 3.0 Criteria [52](#__RefHeading___Toc219020450)

Appendix 11 : IIEF-5 questionnaire [68](#__RefHeading___Toc219020451)

Appendix 12 : The International Prostate Symptom Score (IPSS) [69](#__RefHeading___Toc219020453)

# LIST OF PARTICIPATING CENTERS

| INVESTIGATOR | RESEARCH LOCATION |
| --- | --- |
| **Professor Eric LARTIGAU**  Radiation Oncologist  Principal Investigator:  **Professor Philippe NICKERS**  Radiation Oncologist  Investigator  **Thomas Lacornerie**  **Thierry Sarrazin**  Medical physicists | ***Centre Oscar LAMBRET***  University Radiotherapy Department  3 rue F Combemale  59020 LILLE CEDEX  Tel: (33)3 20 29 59 18 - Fax: (33)3 20 29 59 71  email: e-lartigau@o-lambret.fr |
| **Professor Olivier CHAPET**  Radiation Oncologist  Co-Principal Investigator  **Patrice JALADE**  Medical physicist | ***Centre Hospitalier Lyon Sud***  Department of Radiation Oncology  165 Chemin du Grand Revoyet  69310 PIERRE BENITE  Tel: (33)4 78 86 42 60- Fax : (33)4 78 86 42 65  email: olivier.chapet@chu-lyon.fr |
| **Professor Philippe MAINGON**  Radiation Oncologist  Investigator  **Doctor Gilles CREHANGE**  **Doctor Gilles TRUC**  **Doctor Nicolas BLANCHARD**  Radiation Oncologist  Investigator  **Suzanne NAUDY**  Medical physicist | ***Centre Georges François Leclerc***  Department of Radiation Oncology  1 rue du Professor Marion  21079 DIJON Cedex  Tel: (33)3 80 73 75 17 - Fax: (33)3 80 36 28 29  email: [pmaingon@dijon.fnclcc.fr](mailto:pmaingon@dijon.fnclcc.fr) |
| **Doctor Pascal POMMIER**  Radiation Oncologist  Investigator  **Frédéric GASSA**  Medical physicist | ***Centre Léon Bérard***  Department of Radiation Oncology  28 rue Laennec  6933 LYON Cedex 08  Tel: (33)4 78 78 51 66- Fax: (33)4 78 78 51 40  email: [pommier@lyon.fnclcc.fr](mailto:pommier@lyon.fnclcc.fr) |
| **Doctor David AZRIA**  Radiation Oncologist  Investigator  **Norber AILLERES**  Medical physicist | ***Val d’Aurelle-Paul Lamarque***  208 rue des Apothicaires – Parc Euromédecine  34298 MONTPELLIER Cedex 5  Tel: (33)4 67 61 31 32- Fax: (33)4 67 61 31 35  email: [azria@valdorel.fnclcc.fr](mailto:azria@valdorel.fnclcc.fr) |
| **Professor Didier PEIFFERT**  Radiation Oncologist  Investigator  **Doctor Véronique BECKENDORF**  Radiation Oncologist – Co-Investigator  **Alain NOEL**  **Vincent MARCHESI**  Medical physicists | ***Centre Alexis Vautrin***  6 Avenue Bourgogne Brabois  54500 VANDOEUVRE LES NANCY  Tel: (33)3 83 59 84 31 - Fax: (33)3 83 59 83 91  email: [d.peiffert@nancy.fnclcc.fr](mailto:d.peiffert@nancy.fnclcc.fr) |
| **Doctor Jean-Michel HANNOUN LEVI**  Radiation Oncologist  Investigator  **Gaëlle ANGELLIER**  **Serge MARCIE**  Medical physicists | ***Centre Antoine Lacassagne***  33 Avenue de Valombrose  06182 NICE Cedex 02  Tel: (33)4 92 03 16 51 - Fax: (33)4 92 03 15 70  email**:** jean-michel.hannoun@nice.fnclcc.fr |
| **Doctor Patrice CELLIER**  Radiation Oncologist  Investigator  **Docteur Nathalie NEBOUT**  **Docteur Clothilde MORAND**  Radiations Oncologists  Co-Investigators  **Delphine JARNET**  **Jérôme MESGOUEZ**  Medical physicists | ***Centre Paul Papin***  2, rue Moll  49933 ANGERS Cedex 09  Tél : (33)2 41 35 28 81- Fax : (33)2 41 35 27 35  e-mail **:** [p.cellier@unimedia.fr](mailto:jean-michel.hannoun@nice.fnclcc.fr) |

#

# SYNOPSIS

| SPONSOR | **Centre Oscar LAMBRET**  3, rue Frédéric Combemale  BP 307  59020 LILLE CEDEX  **Tel:** (33)3 20 29 59 18 - **Fax**: (33)3 20 29 59 71 |
| --- | --- |
| INDICATION | Intermediate risk prostate cancer |
| **TITLE** | ***Intermediate risk prostate cancer : Prospective phase II study evaluating hypofractionated stereotactic boost irradiation*** |
| **PRINCIPAL INVESTIGATOR**  **(CyberKnife treatment)**  **Co-PRINCIPAL INVESTIGATOR**  **(LINAC treatment)** | **Professor Eric LARTIGAU**  Radiation Oncology University Department  Tel: (33)3 20 29 59 18 – Fax: (33)3 20 29 59 71  email: [e-lartigau@o-lambret.fr](mailto:e-lartigau@o-lambret.fr)  **Professor Olivier CHAPET**  Radiation Oncology Department  Centre Hospitalier Lyon Sud  69310 PIERRE BENITE  Tel: (33)4 78 86 42 60 – Fax: (33)4 78 86 42 65  email: [olivier.chapet@chu-lyon.fr](mailto:olivier.chapet@chu-lyon.fr) |
| **METHODOLOGY** | Phase II multicentric biomedical research evaluating the feasibility of hypofractionated stereotactic boost irradiation in patients presenting with intermediate risk prostate cancer |
| **NUMBER OF CENTERS** | 8 |
| **OBJECTIVES** | **Primary**   - Evaluate urinary and rectal toxicity according to the NCI-CTCAE v3.0 scale of conventional radiotherapy to 46Gy (prostate and seminal vesicles) followed by a 3 x 6 Gy stereotactic boost (prostate only) administered either with CyberKnife or with a linear accelerator.   **Secondary**   - Evaluate local control at 3 years - Determine the overall survival without metastatic disease - Evaluate survival without biological failure at 3 years - Evaluate technical criteria: implantation of fiducial markers, treatment time, cumulative dosimetry, administration and length of boost session - Study PSA kinetics during treatment course (between the two irradiation phases) and after treatment - Evaluate sexual dysfunction caused by this therapeutic strategy - Evaluate urinary discomfort |
| **INCLUSION CRITERIA** | - Prostate Adenocarcinoma, histologically proven - With at least one of the following criteria : - T2b - and/or PSA between 10 and 20 ng/ml - and/or Gleason Score equal to 7 - Prostate volume ≤ 80 cc - Absence of lymph node involvement (adenopathy < 1.5 cm on scanner or MRI, and/or negative lymph node dissection) - Absence of metastasis (bone scintigraphy) - Age ≥ 18 years - Absence of previous pelvic irradiation - Absence of previous antitumoral treatment, including prostatectomy and/or hormonotherapy - Good general condition: ECOG ≤ 1 (appendix 3) - Absence of contraindication to the placement of fiducial markers – hemostasis problems shall be corrected before implantation - Life expectancy greater or equal to 10 weeks - Patient affiliated with a health insurance system - Patient having signed the informed consent |
| EXCLUSION CRITERIA | - Absence of histological proof - Unfavorable stage (T2c and/or PSA > 20 ng/ml and/or Gleason > 7) - Favorable stage (T1c T2a and PSA < 10 ng/ml and Gleason < 7) - Prior history of uncontrolled cancer and/or treated for less than 5 years (except basocellular skin cancer) - Contraindication to MRI - IPSS score >10 - Recurrent or metastatic disease - Known gold allergy - Patient already included in another therapeutic trial of an experimental drug - Unavailability to medical follow-up for geographical, social or psychological reasons |
| **NUMBER OF PATIENTS** | 76 patients receiving hypofractionated stereotactic boost irradiation :  - 38 by CyberKnife  - 38 by linear accelerator |
| **STUDY DURATION** | Start of the study: September 2009  End of enrollment: September 2012  End of study: December 2015 |
| **ASSESSMENT CRITERIA** | Primary  Urinary or rectal toxicity: According to the NCI-CTCAE v3.0 rating scale (appendix 10). Acute toxicities are defined as all toxicities occurring in the 6 months following the start of radiotherapy. Late toxicities are defined as all toxicities occurring beyond 6 months.  **Secondary**   - Rate of local control at 3 years: non progression of : - PSA level according to the Phoenix definition (nadir to be obtained) - Clinical examination (rectal touch) - No pathological signal on MRI - Median time without progression for the entire cohort: interval between the date of inclusion and the date of progression, whether clinical (rectal touch) or radiological (prostatic MRI). - Median overall survival: Median time for the entire cohort comprising the time between the date of inclusion and the date of death, whatever the cause. - Survival without biological failure at 3 years: Phoenix criteria for PSA increase (nadir + 2 ng/ml) (see chapter 6.7.3) - Technical criteria: Implantation of markers, treatment time, cumulative dosimetry, administration and length of boost session - PSA kinetics during the entire treatment (between the two irradiation phases) and after treatment - Sexual dysfunction = international questionnaire IIEF 5 - Urinary discomfort = IPSS questionnaire |
| **STATISTICAL ANALYSIS** | The initial characteristics of included patients will be presented in the form of summary tables. Continuous variables will be summarized by conventional descriptive statistics parameters (median and outlying  values, mean and standard deviation when necessary). Category variables will be presented in the form of contingency tables (absolute frequency and percentage of each modality), additionally stating the number of missing data, along with the 95% confidence interval.  Subject inclusion and exclusion criteria will be checked against data entered into the case report forms when possible. Any deviations from the protocol will be documented.  Survival will be analyzed by the Kaplan-Meier nonparametric method. |
| **BRIEF DESCRIPTION OF PRODUCTS AND COURSE OF TREATMENT** | - Information provided, collection of consent - Recommended implantation of intra-prostatic markers (fiducials) (see appendix 7 and concomitant treatment 7.6) - Treatment planning - 3D radiotherapy with or without IMRT over 31 to 42 days, 23 x 2Gy/fraction, for a total total of 46 Gy - Stereotactic boost irradiation, 3 x 6 Gy over 5 to 9 days : - delivered by CyberKnife for patients treated in the following centers: Centre Oscar Lambret – Lille, Centre Antoine Lacassagne – Nice, Centre Alexis Vautrin – Nancy - delivered by linear accelerator for patients treated in the other centers |

# 4 INTRODUCTION AND STUDY RATIONALE

## 4.1 Incidence

In France, prostate cancer has become the most frequent cancer in men after age 50 and represents almost 25% of all new cancers in men. The number of cases discovered annually is becoming, in certain regions, superior to the incidence of breast cancer, with an estimated incidence in 2005 of 62,000 according to data from the general cancer registries (Francim). Between 1975 and 2000, an annual increase of 4.8% in the incidence has been noted. The aging of the population cannot completely explain this net increase, which is concomitant with the widespread use of PSA testing and the diffusion of systematic echo-guided techniques of prostate biopsy (120 to 150,000 prostate biopsies performed in France annually). The median age at diagnosis is decreasing (currently around 70 years); it was 74 years in 1995. The probability that a French male will be discovered to have prostate cancer during the course of his life was estimated at 7.9% in 1992 and at 13% in 1998 (i.e., 1 man in 8 less than 75 years old).

## 4.2 Mortality

Prostate cancer is the second leading cause of death by cancer in men after lung cancer and the 4^th^ overall cause of death by cancer. With 10,004 deaths in 2000, it represents 10% of cancer deaths in men (crude death rate: 35.1/106 men). The mortality rate from prostate cancer has increased by 0.17% per year from 1980 to 2000, but the study undertaken between 1990 and 1996 demonstrated that the mortality rate had decreased by 3.3% per year in five French departments, with a stable mortality adjusted by age since 1980. The risk of developing prostate cancer is increasing for younger age groups. For a man born in 1928, the risk of developing prostate cancer before 75 years (cumulative rate) is estimated at 7.3%. This risk is multiplied by 2.97 for a man born in 1943 and almost by 5 for a man born in 1953, if the current trend continues.

In 1995, the French incidence rate was slightly above the mean of European Union countries (after Finland, Sweden, Holland, Belgium, Ireland, Germany and Austria). The mortality rate remains relatively homogenous among these countries. Mortality seems to decrease in some countries (Canada, United Kingdom, Italy, United States, Austria), without being explained solely by screening (not done in the UK). Only the results (expected in 2 to 4 years) of randomized screening studies will allow a conclusion on the effect of screening in regards to mortality.

## 4.3 Study Justification

### 4.3.1 Radiotherapy for localized prostate cancer

Radiotherapy has demonstrated curative efficacy in prostate cancer at the localized stage [Bey 2002, Soulié 2004]. Conformal radiotherapy [Dubray 1999] relies on CT imaging in order to precisely identify the anatomic location of target volumes (prostate, seminal vesicles) and healthy organs (rectum, bladder, femoral heads). Computerized tools for the reconstruction of images - “views of the treatment beam” - allow individualized adaptation of the shape of the beam in order to optimize irradiation ballistics. The contribution of different beams is then confirmed during preliminary dosimetric planning, using computer programs to calculate the distribution of expected doses in a three dimensional representation of the patient.

Two randomized studies have demonstrated that conformal irradiation decreased early and late digestive toxicity [Dearneley 1999, Koper 2004]. This benefit is the result of a better delineation of the prostate and conformality of the beam and consequently a reduction of the volume of irradiated healthy tissue. The total recommended doses are from 70 Gy in 35 sessions of 2 Gy for low risk cancers, and at least 74 Gy (37 sessions of 2 Gy) for intermediate or higher risk cancers [Soulié 2004].

### 4.3.2 Hypofractionated Radiation Therapy

An abundant clinical and experimental literature has demonstrated that the administration of doses superior to 2 Gy per session significantly increases the risk of late irradiation sequelae [partial review in Brenner 2004]. Therefore, the dose is usually of 2 Gy per fraction and the consequence is a radiation therapy treatment lasting 2 months (35 to 40 fractions, 5 fractions per week). Considering the total dose to administer, patients with prostate cancer are having radiotherapy proposed to them over almost 2 months (35 to 40 sessions at a rate of 5 sessions per week).

Doses superior to 2 Gy per session (hypofractionated radiotherapy) are traditionally used for palliative treatments, or for patients whose life expectancy is short. However, since the end of the 1990s, several publications have raised interest in delivering higher dose per fraction, (hypofractionated radiotherapy) for prostate cancer.

Analysis of patient populations treated with external beam therapy or brachytherapy with the help of a mathematical model integrating the impact of fractionation has suggested that doses per session greater than 2 Gy would increase antitumoral efficacy [Brenner 2002, Fowler 2003, Bentzen 2005]. Following the example of healthy tissues, repair of radio-induced lesions would be less effective in prostate cancers than in the majority of tumors, hence a greater biological efficacy of hypofractionated irradiation.

Based on these recent data, some teams [Junius 2007, Livsey 2003, Yeoh 2003] have published promising results in terms of efficacy and tolerance.

The development of radiotherapy under conformal conditions allows better protection of healthy tissues, and so probably the reduction of the harmful effects of hypofractionated radiotherapy.

### 4.3.3 Importance of the irradiation total dose

Five randomized prostatic studies report an improvement in the rate of biological control at 5 years with an increase dose of irradiation without a major increase in the complication rate (Albertsen 1996, Bey 2002, Amer 2003, Beckendorf 2004, Bentzen 2005). These studies are as much on conformal radiotherapy with or without intensity modu-lation as on high or low dose rate brachytherapy (Bey 2002). The brachytherapy boost techniques are close to ablative treatments, as demonstrated by a very rapid drop in PSA levels and the rapid normalization of spectroscopic examinations compared to external beam radiotherapy alone (Brenner 2004).

With a long enough follow-up, the relation between biochemical control, decrease of distant spread, and perhaps survival appears clearer and clearer (Brenner 2002, Chirpaz, 2002, Dearnaley 1999, Dubray 1994-1999). However, it is possible that the high doses currently administered are still not high enough to obtain the highest rates of biochemical control considering the presence of residual hypoxic zones in the prostate (Fayers 1995). Additional benefit could be obtained with higher doses with a biological equivalence (EQD2 of 100 Gy for a α/β of 3Gy (AFU 2002 guidelines). Consequently it is probable that we have not yet reached the optimal dose levels with current techniques of prostate radiotherapy, although numerous technological improvements are still possible.

### 4.3.4 Stereotactic radiation therapy concept

The concept of stereotactic radiotherapy consists of delivering a high irradiation dose highly conformal to a small tumor. The result is an “ablation” of the tumor, while preserving the surrounding tissues. This technique requires high precision in the localization of the tumor, and for this reason, was first developed for the treatment of cerebral metastases. Indeed, in immobilizing the skull (and therefore the brain), it is possible to localize an intra-cerebral lesion with precision. Local control results have allowed stereotactic radiotherapy to be proposed as an alternative to surgery in the treatment of cerebral metastases. More recently, the utilization of new technologies in the spatial localization of tumors, such as Cone Beam CT, has allowed the development of this irradiation technique in extra-cranial tumors. The Cone Beam CT allows the acquisition of scanography slices directly on the accelerator table while the patient is already in the treatment position. It has become possible to precisely visualize tumor’s position, even in soft tissues (lung, liver, prostate…). Thus, stereotactic radiotherapy has been used in the irradiation of small size lesions of bronchial cancers (T1 and T2N0). For these tumors, although considered radio-resistant, the local control rate has raised from 30%-40%, after conventional radiotherapy, to 80%-90% after stereotactic radiotherapy (Timmerman 2003 & 2006, Fritz 2006, Nyman 2006, Xia 2006, Hara 2006).

Stereotactic radiation therapy has been developed in other tumoral localizations such as liver (Tse 2008), kidney (Swedman 2008) or prostate.

### 4.3.5 Stereotactic radiotherapy of prostate cancer

Some work has been done on stereotactic radiotherapy for prostate cancer. However, few studies have evaluated this technique either as monotherapy or as a boost to conventional fractionated radiotherapy. Madsen and al reported 40 patients treated with monotherapy stereotactic radiation treatment with a dose of 33.5Gy in 5 fractions [Madsen 2007]. Irradiation was administered with a linear accelerator. The equivalent biological dose was estimated at 78Gy. No late urinary or rectal toxicity was observed and only one acute grade 3 intestinal toxicity was observed. Authors had previously evaluated prostate intra-fraction motion [Madsen 2003]. Following a low-gas, low-motility diet, mean movements were of 2 mm, 1.9 mm and 1.4 mm in the superior-inferior, anterior-posterior and right-left directions respectively. In a similar study, King and al have evaluated the feasibility of CyberKnife stereotactic radiation treatment as monotherapy for localized prostate cancer at a total dose of 36.7Gy in 5 fractions of 7.25Gy each. Only 2 late urinary toxicities of grade 3 were reported. Authors emphasized the necessity of a minimum of 1 day between each fraction in order to prevent severe rectal toxicities. Mantz and al followed the same irradiation protocol in 18 patients with an adenocarcinoma at a favorable stage. The prostatic volume was limited to 60 cc. Irradiation was administered with a Trilogy system (Varian). After few weeks/months of follow-up, only grade 1 rectal toxicities were reported.

Finally, a stereotactic irradiation of prostate (5 x 9 Gy) is on going at Dallas University. Intra-prostatic markers and an endo-rectal balloon are used [Edwards 2007]. Intra-fraction mean prostatic movements are of 2 mm in all directions [Boike 2007].

A second approach is to propose a stereotactic radiotherapy as a boost to conventional irradiation for intermediate risk prostate cancer. Boosts are already delivered by HDR brachytherapy for low or intermediate risk prostate cancer. Usually, 36 to 50Gy are administered on the prostate and the seminal vesicles followed by 3 to 4 brachytherapy sessions of 5 to 6Gy [Hoskin 2007-2008, Demanes 2005, Galalae 2004, Eulau 2000]. Doses delivered at the level of the prostate are higher than the dose delivered with external beam radiation therapy alone. Results are very promising particularly biological control with no significant increase of rectal and urinary complications. However, HDR brachytherapy remains an invasive procedure requiring anesthesia. Stereotactic radiotherapy could potentially deliver doses equivalent to HDR brachytherapy in a non-invasive manner and without anesthesia. Ares and al studied the incidence of late rectal toxicities of a boost delivered either by HDR brachytherapy or stereotactic irradiation [Ares 2007]. Following 3D external beam irradiation to 64-66Gy, a brachy-therapy boost (2 x 5 to 8Gy) was adminisred to 51 patients (Geneva University) and a stereotactic radiotherapy boost was delivered to 43 patients (Barcelona University). No grade 3 rectal toxicity was observed. Grade 1-2 rectal toxicity was slightly higher with stereotactic radiotherapy than with HDR brachytherapy. However, compared to 3D radiotherapy to 74-78Gy, the rate of grade 1-2 rectal toxicity are clearly lower.

The objective of this project is to evaluate the tolerance of high doses by fraction, administered in a stereotactic mode as a boost to external beam therapy in patients with intermediate risk prostate adenocarcinoma [Kattan 2000]. Given the exclusion of unfavorable-risk tumors, hormonotherapy will not be introduced, based on the lack of benefit after high radiation doses for this category of patients [Kattan 2000, Eade 2007]. The goal of this study is also to confirm the data of the William Beaumont Hospital, showing that with an EQD2 of 100 Gy (for an α/β of 3 Gy), a very high level of disease control can be obtained, with a possible impact on overall survival. This approach will also study the kinetics of PSA decrease, indirect indicator of the ablative effects of the irradiation methods, in order to compare them to brachytherapy [Koper 2004].

Stereotactic treatments can be administered with modern radiation therapy equipments. The CyberKnife system, which uses robotics, can deliver treatment with inframillimetric precision and real time tracking of the target. Linear accelerators can entirely deliver precise treatments with the possibility of intensity modulation

These technological advances allow reduction of the dose to healthy tissues surrounding the treatment area and limit the margins traditionally required in order to take movement into account. It therefore becomes possible to deliver a very high dose without major risk of toxicity [Hannoun-Levi 2008].

***Therefore, we propose a phase II multicentric study evaluating the feasibility of external beam radiotherapy at 46Gy combined with boost irradiation by stereotactic radiotherapy (CyberKnife or linear accelerator) in patients presenting with intermediate risk adenocarcinoma of the prostate, with the object of delivering an equivalent biological dose on the order of 80Gy while best preserving critical organs.***

# 5 STUDY OBJECTIVES

## 5.1 Primary objective

- Evaluate urinary and rectal toxicity according to the NCI-CTCAE v3.0 scale of conventional radiotherapy to 46Gy (prostate and seminal vesicles) followed by a 3 x 6Gy stereotactic boost (prostate only) administered either with CyberKnife or with a linear accelerator

## 5.2 Secondary objectives

- Evaluate local control at 3 years
- Determine the overall survival without metastatic disease
- Evaluate survival without biological failure at 3 years
- Evaluate technical criteria: implantation of fiducial markers, cumulative dosimetry, administration and length of boost session
- Study PSA kinetics during treatment (between the two irradiation phases) and after treatment
- Evaluate sexual dysfunction caused by this therapeutic strategy
- Evaluate urinary discomfort

# 6 STUDY DESIGN

## 6.1 Methodology

Phase II multicentric biomedical research evaluating the advantages of hypofractionated stereotactic boost irradiation, done by CyberKnife or linear accelerator, in patients with intermediate risk prostate cancer

## 6.2 Inclusion Criteria

*All the following criteria are necessary for inclusion :*

- Prostate adenocarcinoma, histologically proven
- With at least one of the following intermediate criteria :
- T2b
- and/or PSA between 10 and 20 ng/ml
- and/or Gleason Score equal to 7
- Prostate volume ≤ 80 cc
- Absence of lymph node involvement (adenopathy < 1.5 cm on scanner or MRI, and/or negative lymph node dissection)
- Absence of metastasis (bone scintigraphy)
- Age ≥ 18 years
- Absence of previous pelvic irradiation
- Absence of previous antitumoral treatment, including prostatectomy and/or hormonotherapy
- Good general condition: ECOG ≤ 1 (appendix 3)
- No contraindication to fiducial markers placement– hemostasis problems shall be corrected before implantation
- Life expectancy greater or equal to 10 weeks
- Patient affiliated with a health insurance system
- Patient having signed the informed consent

## 6.3 Exclusion criteria

*One of the following criteria is sufficient for exclusion:*

- Absence of histological proof
- Unfavorable stage (T2c and/or PSA > 20 ng/ml and/or Gleason > 7)
- Favorable stage (T1c T2a and PSA < 10 ng/ml and Gleason < 7)
- Prior history of uncontrolled cancer and/or treated less than 5 years (except basocellular skin cancer)
- Prostate cancer previously treated with hormonotherapy
- Contraindication to MRI
- IPSS score > 10
- Recurrent or metastatic disease
- Known gold allergy
- Patient already included in another therapeutic trial of an experimental drug
- Unavailability to medical follow-up for geographical, social or psychological reasons

## 6.4 Early study withdrawal criteria

The length of patient participation in the study is at least 3 months after the last radiotherapy session.

The conditions for early termination of the study are :

- Failure to track the tumor
- Failure to place fiducial markers
- Dose-volume histograms (DVH) constraints not achievable
- Interval greater than 3 weeks between the end of the radiation therapy phase and the first session of hypofractionated stereotactic boost irradiation

## 6.5 Termination study criteria

Termination from the study must take place if:

- disease progression

- patient’s decision

- investigator’s decision

- death of the patient

## 6.6 Assessment visit

The summary table is described in appendix 2.

### 6.6.1 Inclusion examination

Patients eligible for the trial and having signed their consent to participate shall have an initial assessment within the 21 days preceding the treatment start.

 Clinical examination

- Weight, height, performance status or WHO (appendix 3)
- Digital rectal exam
- Cardiovascular risk factors (HBP, hypercholesterolemia, diabetes, stress)
- Prior medical and surgical history
- Disease history
- Tobacco use

 Blood tests

- CBC - Platelets

- Prothrombin rate, TCA, INR

- Urea, creatinine

- Tumor marker: PSA

 Paraclinic tests

- Tumor evaluation by pelvic spiral CT (could be the planning scanner) and pelvic and prostatic MRI
- Planning CT-Scan (1)
- Bone scan

 Questionnaires

- Sexual quality of life “IIEF-5” (appendix 11)

- Urinary difficulty “IPSS” (appendix 12) score >10 is an exclusion criteria

 Disease classification

- Gleason score, number of positive biopsies and total length of positive biopsies (appendix 5)

1. *For dosimetry, the planning scanner will be conducted with the most informative sequences identified by the diagnostic scanner and fused with the MRI.*

### 6.6.2 Evaluation during treatment

Evaluation of tolerance according to the NCI-CTC AE v.3.0, weekly during conformal radiotherapy (first treatment phase) then before each stereotactic radiotherapy session.

***The following evaluation will be done before starting the hypofractionated stereotactic boost :***

 Clinical examination

- Weight, performance status or WHO (appendix 3)
- Digital rectal exam

 Blood tests

- Urea, creatinine
- Tumor marker : PSA

### 6.6.3 Post-treatment visit

Evaluation will be done 3 months after the last radiotherapy session :

 Clinical examination

- Weight, performance status WHO (appendix 3)
- Digital rectal exam
- Evaluation of acute toxicity related to irradiation

 Blood tests

- Urea, creatinine

- Tumor marker: PSA

 Paraclinic examination

- Prostate and pelvis MRI

 Questionnaires

- Sexual quality of life “IIEF-5” (appendix 11)

- Urinary discomfort “IPSS” (appendix 12)

### 6.6.4 Follow-up visit

Evaluation will be done 6, 9, 12, 18, 24 and 30 months after the last radiotherapy session :

 Clinical examination

- Weight, performance status or ECOG (appendix 3)
- Digital rectal exam
- Evaluation of late toxicity associated with irradiation

 Blood tests

- Tumor marker: PSA

 Paraclinic examinations

- Prostate and pelvis MRI (at 6, 12 and 24 month)

 Questionnaires

- Sexual quality of life “IIEF-5” (appendix 11)

- Urinary discomfort “IPSS” (appendix 12)

### 6.6.5 End of study evaluation

Evaluation will be done 36 months after the last radiotherapy session or at premature end from the study (see chapter 6.5).

 Clinical examination

- Weight, performance status or ECOG (appendix 3)
- Digital rectal exam
- Evaluation of late toxicity associated with irradiation

 Blood tests

- Tumor marker: PSA

 Laboratory and special studies/tests

- Prostate and pelvis MRI

 Questionnaires

- Sexual quality of life “IIEF-5” (appendix 11)

- Urinary difficulty “IPSS” (appendix 12)

### 6.6.6 Long term evaluation

Long term evaluation will be identical to the follow-up evaluation (see chapter 6.6.4) and will be done 48 and 60 months after the last radiotherapy session.

## 6.7 Assessment Criteria

### 6.7.1 Tolerance evaluation

Acute tolerance (≤ 6 months from the end of radiotherapy) and late effects associated with radiotherapy (> 6 months, from the end of radiotherapy) will be assessed according to the NCI-CTCAE scale, version 3.0 (appendix 10), from the data collected by the clinician.

#### Non-serious adverse events

Adverse event refers to any change in the initial condition of the patient prior to treatment, including intercurrent diseases, whether the event is considered as being related to irradiation or not and occurring at any time during the study.

The nature of each adverse event is established, along with the date of occurrence, duration, severity, relationship to treatment, associated treatments and outcome. The intensity of adverse events will be graded according to the criteria defined for the disease studied (NCI-CTCAE version 3.0 criteria – appendix 10).

#### Serious Adverse Events

Definition:

Not considered as a serious adverse event (SAE):

- Hospitalization < 24 hours (medical imagery examination, blood test, for example…)
- Planned hospitalization prior to the start of the trial and/or planned in the protocol

*The term serious adverse event (SAE) refers to any event:*

- Leading to death,
- Life threatening,
- Leading to hospitalization, or prolongation thereof,
- Causing permanent disability or serious temporary impairment,
- Causing a congenital abnormality, fetal malformation, or abortion,
- Medically significant.

The terms *disability* and *impairment* correspond to any clinically significant temporary or permanent physical or psychological handicap impacting the patient's physical activity and/or quality of life.

Any clinical event or laboratory result is considered *medically significant* if it is considered serious by the investigator and does not correspond to the above-defined seriousness criteria. These may represent a risk for the patient requiring medical intervention to prevent an outcome corresponding to one of the previously mentioned seriousness criteria *(*e.g., *overdosages, secondary cancers, and new facts that may be considered medically significant).*

#### *Serious adverse event - expected (SAE-E)*

A SAE-E is an event already mentioned in the results of studies with this type of treatment :

- Rectitis +/- hemorrhagic
- Cystitis +/- hemorrhagic

#### *Serious adverse event - unexpected (SAE-U)*

A SAE-U is an event other than those mentioned above or different in its nature, intensity, or development in regards to this list.

#### Intensity criteria

The intensity criterion must not be confused with the seriousness criterion, which serves as a guide to define declaration obligations.

The intensity of events will be estimated according to the NCI-CTCAE version 3.0 classification (toxicity grade 1 to 5). The intensity of unlisted adverse events in this classification will be estimated according to the following qualities :

**Mild** (grade 1): does not affect the patient's daily activity

**Moderate** (grade 2): disturbs the patient's daily activity

**Severe** (grade 3): prevents the patient's daily activity

**Very severe** (grade 4): requires reanimation measures / life threatening

**Death** (grade 5):

#### Steps to take

The investigator informs the Integrated Clinical Research Unit of all **Serious Adverse Events** whether or not they can be attributed to the research, which occur during the study or in the 3 months following the first irradiation session.

All late Serious Adverse Events (occurring after this 3 month period) considered as being reasonably related to the protocol treatment or to the research, must be declared without time limitation.

Declarations will be submitted by fax to the Clinical Research Unit using the "***serious adverse event notification***" form (appendix 10), documented in the most precise manner possible, dated and signed, within **24 working hours** of their observation, to :

#### Tel: 03 20 29 59 18 - Fax: 03 20 29 59 71

The investigator must also, whenever possible, attach the following documents to the serious adverse event report:

- A copy of the hospitalization record, or hospitalization prolongation record,
- A copy of the autopsy report,
- A copy of the results of all additional examinations performed, including any pertinent negative results, appending the laboratory's normal values.
- Any other documents deemed useful and relevant.

All of these documents must be anonymous.

Additional information may be requested (by fax, phone, or during a visit) by the monitor.

#### SAE monitoring

The investigator is responsible for providing appropriate medical monitoring of patients until the event is resolved or stabilized, or until the patient's death. **This may occasionally require monitoring after the patient's exit from the trial.**

He/she transmits the additional information to the Clinical Research Unit by means of an SAE declaration form (ticking the Follow-up N° X box to state that it is a follow-up and not an initial report) within 24 hours of receiving it. He/she also transmits the last follow-up on resolution or stabilization of the SAE.

He/she keeps all relevant documents concerning the presumed adverse event in order to be able, if necessary, to complete any previously transmitted information.

He/she responds to any additional information requests from the Clinical Research Unit's PV in order to document the initial observation.

6.7.2 Evaluation of tumor efficacy

To be deemed assessable, patients must have received **at least 1 session of boost stereotactic irradiation**.

#### *Local control at 3 years of treatment*

Local control will be defined by :

- the non-progression of PSA according to the Phoenix criteria after obtaining the nadir,

- a non-progressive clinical exam (digital rectal exam),

- and the absence of pathologic signal with MRI.

#### *Global survival*

Time between :

- inclusion date,
- date of death, whatever the cause,
- Patients alive at the time of the analysis will be censored at the date of the last follow-up visit.

#### *Survival without metastatic disease*

Time between:

- inclusion date,
- date of appearance of the first metastasis.

### 6.7.3 Evaluation of biological response

The Prostate Specific Antigen (or PSA) is sufficiently specific of prostate adenocarcinoma. The biological response will be evaluated according to the progression of PSA, measured at each monitoring visit.

Biological progression is defined as:

- according to the Phoenix definition, biological progression is defined as an increase in the total PSA serum level + 2 ng/ml above the nadir

- Recommendation : in case of PSA elevation of less than nadir + 3 ng/ml, it is not recommended to plan a specific salvage therapy (Phoenix expert panel consensus [Roach 2006])

- In case of death, the patient is considered in biological remission if a PSA increase has not been confirmed by three successive measures.

### 6.7.4 Evaluation of technical criteria

- Implantation of fiducial markers : Yes / No
- Cumulative dosimetry : single/dual
- Boost session done : Yes / No
- Length of the boost session : Time elapsed between the patient entry and exit from the treatment room (bunker)

### 6.7.5 Evaluation of PSA kinetics during treatment

PSA will be obtained in the 48 hours following the last irradiation session of the first treatment phase will be compared to PSA before treatment, at the end of the treatment and then on a quarterly basis. PSA progression between these two samples will be correlated with the local control post-treatment.

### 6.7.6 Evaluation of sexual dysfunction

The International Index of Erectile Function is a questionnaire containing 15 questions, whose response is checked from 0 to 4 or 5 per question. It allows a semi-quantifiable evaluation of different aspects of masculine sexuality, grouped under the “areas” of erection, satisfaction, orgasm and desire. It was developed in the USA by Prof. Rosen and has since been translated and validated in numerous languages, including French [Rosen 1997]. A simplified questionnaire containing only 5 questions, the IIEF 5, has been extracted for “screening” for erectile problems (appendix 11) [Rosen, 1999]. The complete version, meant for research and in particular the objective evaluation of treatment results, will be used in this study.

Interpretation will be a function of the number of points scored :

- 5 to 10 Severe erectile dysfunction

- 11 to 15 Moderate erectile dysfunction

- 16 to 20 Mild erectile dysfunction

- 21 to 25 Normal erectile function

- 1 to 4 Non interpretable

### 6.7.7 Evaluation of urinary discomfort

The IPSS (*International Prostate Score Symptom)* is a self-administered questionnaire whose French version has been validated (appendix 12). The IPSS is made of 7 questions evaluating the severity of urinary symptoms. The questionnaire has been developed for the patient’s self-evaluation. Responses are checked from 0 to 5. Each question has six possible responses quantifying in increasing fashion the severity of symptoms. The total score can consequently be added, from 0 to 35 points (asymptomatic to very symptomatic). Patients can be classified as follows:

0-7: few symptoms

8-19: moderate symptoms

20-35: severe symptoms

##

## 6.8 Statistical analysis and determination of the number of patients

### 6.8.1 Data management

Data input and management shall be performed by the Centre Oscar Lambret Biostatistics Unit. A study-specific database shall be created, tested and validated before data entry. This data base shall be developed by the “Data Treatment Center of the North-West Canceropole, authorized by the INCa” with the Capture System (CLINSIGHT). This software program has been designed for the global management of clinical studies, and meets the regulatory requirements associated with this type of study. A data validation plan shall be defined, providing a detailed description of the controls required for each variable, along with the list of obvious corrections allowed.

Questionnaires shall be validated on receipt and shall be independently entered twice. After comparing the two entries and generating a single database, the data shall be checked by the team in charge of data management using the error messages generated by the validation programs. Any obvious errors shall be corrected. Other errors, omissions or inconsistencies shall be stated on correction request forms that shall be sent to the investigating physician for resolution. Once the Biostatistics Unit receives the investigating physician's response, the corrections shall be added to the database.

The database is locked after final quality control, then exported to the SPSS statistical software using an automated and validated procedure.

### 6.8.2 Statistical analysis

The initial characteristics of included patients will be presented in the form of summary tables. Continuous variables will be summarized by conventional descriptive statistics parameters (median and outlying values, mean and standard deviation when necessary). Category variables will be presented in the form of contingency tables (absolute frequency and percentage of each modality), additionally stating the number of missing data, along with the 95% confidence interval.

Subject inclusion and exclusion criteria will be checked against data entered into the case report forms when possible. Any deviations from the protocol will be documented.

Survival will be analyzed by the Kaplan-Meier nonparametric method.

### 6.8.3 Determination of the number of subjects

The number of patients required for this study was calculated using the single step Fleming method with the following hypotheses :

P_0_ = 85% (level of patients without any toxicity below which the method would provide no

advantage)

P1 = 95% (level of patients without toxicity expected from the proposed methods)

α= 5%

β= 10% , i.e. a power of 90%

To meet these conditions, the Fleming formula requires 76 evaluable patients to be included (38 by CyberKnife and 38 by linear accelerator). The study's goal will be considered achieved if, of the 76 evaluable patients, at least 70 present no grade 3-4 toxicity according to the NCI scale.

### 6.8.4 Criteria for termination of the study

The study will be terminated if :

- a Grade 4 toxic event is observed according to the NCI scale,
- or a death in the 3 months following the last session of hypofractionated stereotactic boost irradiation

**in 2/3 or 3/8 or 4/11 or 5/18 patients.**

# 7 TREATMENT

## 7.1 Patient enrollment

An inclusion request form will be completed by the investigator in order to ensure that the patient fulfils ALL selection criteria. The investigator must provide the information letter to the patient and have him sign the consent form making explicit its free and informed consent.

BEFORE INITIATING TREATMENT, the investigator must fax the inclusion request form to the Coor-dination Center of the study:

Clinical Research Integrated Unit / Promotion Section

Centre Oscar Lambret – Lille

#### Tel: 03 20 29 59 18 - Fax: 03.20.29.59.71

After verification of all selection criteria, an identification number shall be allocated to the patient and shall be maintained throu-ghout the trial. The identification number shall be faxed to the investigator. ***Investigator : Monitor :***

**Professor Eric LARTIGAU Muriel CHAPOUTIER**

Tel: 03 20 29 55 98 Tel: 03 20 29 55 68

Fax: 03 20 29 59 71 Fax: 03 20 29 59 71

email: [e-lartigau@o-lambretr.fr](mailto:e-lartigau@o-lambretr.fr) email: [m-chapoutier@o-lambret.fr](mailto:m-chapoutier@o-lambret.fr)

## 7.2 FIRST PHASE : Conformal 3 D radiotherapy with or without intensity modulation

23 fractions, 2Gy per session, will be delivered over a maximum of 42 days for a total dose of 46Gy (see appendix 1).

### 7.2.1 Patient positioning

Patient set-up is left free, as is the immobilization technique. However, set-up reproducibility within 5 mm must be verified weekly during treatment. The patient should be advised to have a rectal evacuation, if possible, before scanning and before each treatment session. A certain degree of bladder filling should be maintained (last miction 1 hour before CT-Scan and/or irradiation treatment followed by a glass of water).

### 7.2.2 Preparation for treatment – Placement of intra-prostatic markers (fiducials)

Intra-prostatic markers shall be implanted before examination by MRI or scanner. The choice of markers shall be left to the discretion of each participating center (gold seeds, …). These shall insure precise repositioning of the prostate (≤ 2 mm). They will also allow a precise fusion between the MRI and the dosimetric scanner.

This placement is recommended.

#### Fiducial markers

These are markers (gold seeds…) allowing the tracking of mobile lesions.

Treatment preparation includes the following steps :

- Consultation with the radiation oncologist
- Blood tests : PT, TCA, Platelets
- Placement of fiducial markers by a radiologist under ultrasound control (fiducial placement requirements and guidelines, appendix 7, see chapter 7.6 concomitant treatment)
- individualized immobilization system
- at least one week after fiducial placement: completion of planning scanner (recommendations appendix 7)
- dosimetry

### 7.2.3 Images acquisition

An MRI and scanner are done for the first treatment phase. CT-scan image acquisition is done in the treatment position, with the immobilization accessories. An intravenous injection of contrast agent must be given, in the absence of contraindication. Images must allow visualization of the anatomical structures, and also the markers implanted in the prostate. The bladder will be in intermediary state (neither full nor empty: last miction 1 hour before followed by a glass of water) and the rectum will be empty and non-distended.

CT-scan slices are up to 3 mm and obtained between the L5-S1 and the lesser trochanters.

After image transfer to the planning system, fusion with the prostatic MRI will be done, for a better definition of prostate contours, in particular the apex.

### 7.2.4 Identification of target volumes

CTV1 (first time of treatment) will include prostate and seminal vesicles.

- **Prostate** : The contour includes the entire gland. The definition of the apex is sometimes difficult : frontal and sagittal recontuctions (1 cm above the prostatic bulb) and image fusion with MRI. If doubt, especially if the apical biopsies are positive or if the lesion is situated low on the MRI, additional cuts will be added near the bottom. The principal anticipated toxicity of an irradiation administered too low is alteration of sexual function. The base is identified with the help of vesicular opacification
- **Seminal vesicles:** only the proximal part of the seminal vesicles will be included in the CTV1.

### 7.2.5 Localization of the prostate

If conformal radiotherapy with intensity modulation is used for the first treatment phase, daily repositioning of the prostate on the implants or with the Conebeam CT is requested.

### 7.2.6 Dosimetry

Four to seven beams will be used with energy photons between 6 and 21 MV. PTV1 is defined by 1 cm expansion of CTV1, margin reduced posteriorly to 0.5 cm in order to protect the rectum. Beams limits are defined by additional margin around the PTV. The dimensions of this margin are a function of the penumbra of the beam (ICRU 50).

We will strive to respect ICRU 50 recommendations (doses received at each point of the PTV must be superior to 95% and less than 107% of the prescribed dose). In order to respect the constraints on the dose received by healthy tissues, the minimal dose within the target volume is recognized to be less than the prescribed dose. We will strive to have the minimal dose within the PTV to be at least 95% of the prescribed dose. Dose prescription is made at ICRU point (conformal radiotherapy). If case of IMRT, the mean dose to the PTV1 would be at least equivalent to prescribe dose. In all cases, heterogenity will be considered.

### 7.2.7 Doses to critical organs

#### Contouring organs at risk

**Rectum and anal canal :**

- Lower limit : 2 cm below the CTV1
- Upper limit : at the junction of the first sigmoid loop
- The rectal volume is the volume included between the external contour and an internal contour of 5 mm (“dose-wall histogram: DWH”)

**Bladder :**

- External contouring of the entirety from slices where it is visible. The bladder volume is the volume included between the external contour and an internal contour defined at 7 mm from the former
- When the prostate sticks out into the bladder, it will be necessary to ascertain that there is not an overlap between the critical organs and the CTV.

**Intestine and sigmoid :**

- We will strive to quantify in one or two separate critical volumes the small intestine and the sigmoid

**Penile bulb :**

- Drawn on the cuts where it appears

#### Dose constraints

Summarized in the table below :

| **Rectum** | **Bladder** | **Femoral heads** |
| --- | --- | --- |
| <50 Gy in 5% of the volume | <50 Gy in 5% of the volume | Maximum point <30 Gy |
| <47 Gy in 30% of the volume | - |
| <40 Gy in 40% of the volume | <40 Gy in 50% of the volume |

## 7.3 SECOND PHASE : Hypofractionated stereotactic boost irradiation

**Hypofractionated stereotactic boost irradiation begins in the 10 days following the last session of the first phase.**

The 3 fractions, at 6 Gy each, are delivered over 5 to 9 days (typically Tuesday, Friday and Tuesday). An interval of 48 hours will be observed between two sessions of stereotactic radiotherapy delivered by:

- linear accelerator for patients treated in the following centers: CH-Lyon Sud, Centre GF Leclerc-Dijon, Centre L Bérard-Lyon, Val d’Aurelle-Paul Lamarque-Montpellier, Centre Paul Papin-Angers
- CyberKnife for patients treated in the following centers: Centre O Lambret-Lille, Centre A Lacassagne-Nice, Centre A Vautrin-Nancy

### 7.3.1. By linear accelerator

#### Patient positioning

The patient will be in dorsal decubitus, in a stable position reproducible session after session. Different immobilization systems can be used, including a stereotactic frame with vacuum mattress. The immobilization of the patient must be sufficiently reliable in order to ensure that the Clinical Tumor Volume (CTV2) does not exceed the Planning Tumor Volume (PTV2).

#### Image acquisition

Iin case of different contention or modification in patient setting, new image acquisition for planning purposes will be acquired at the discretion of the clinician according to the same guidelines described for phase 1. MRI may be done if a rectal-balloon is placed.

#### Volume definitions

The prostate without the seminal vesicles will represent the Clinical Target Volume (CTV2). Prostate contouring will be done by a radiation oncologist experienced in the definition of prostatic volumes on scanner and MRI. MRI contours will be fused with the scanner contours in order to better define the prostatic apex.

The PTV2 will be obtained by an expansion of 5 mm around the CTV2 according to Boike and al data [Boike 2007].

The prostatic urethra will be delineated on MRI from the bladder up to 2 cm below the apex of prostate. A 3 mm margin will be defined around the urethra.

The rectum will be delineated with an internal expansion of 5 mm from 2 cm below the prostatic apex (without differentiation rectum / anal canal) up to the recto-sigmoid junction.

The empty bladder will be delineated in its totality with an internal expansion of 7 mm.

The femoral heads will be delineated up to the superior pole of the lesser trochanter.

#### Control of prostate movements

Approximately 30 minutes before the planning scanner and at each treatment session, an enema will be administered in order to evacuate matter and gas. An endo-rectal balloon may be placed to limit movement and ensure a better repositioning of the prostate. In order to avoid the transmission of the respiratory movements to the prostate, the bladder will be empty. Movement control can be done either by radioscopy on intra-prostate implants, or by 4D scano-graphy acquisition. The latter method should be used whenever available. In certain cases prostate movements will be limited and will not require immobilization. If prostate movements are greater than 5 mm, abdominal compression, gating or breath holding will be used to limit movements. If, despite that, prostate movements remain greater than 5 mm, stereotactic boost irradiation cannot be done, and irradiation will be accomplished according to conventional methods.

#### Prostate localization

Intra-prostatic markers will be implanted in the prostate before the first scanner acquisition of phase 1. The choice of markers will be left to the discretion of each participating center (gold seeds,…). These must ensure precise repositioning of the prostate (≤ 2 mm). They will also allow a precise fusion between the MRI and the planning scanner. At each stereotactic irradiation session, a precise repositioning (less or equal to 2 mm) of the prostate will be ensured by Conebeam CT acquisition or by systems like Exact Trac. Between two irradiation beams, additional checks can be done in doubtful cases by the acquisition of orthogonal X-ray, the use of a Snap Verification system, or even a new Conebeam CT. If these checks are frequent, the additional dose received must be subtracted from the planned irradiation dose.

#### Dosimetry

The beams will be produced by a linear accelerator with photon energy between 6 and 21 MV. 10 to 15 coplanar or non-coplanar beams will be necessary with 3D dosimetry and, if necessary, the use of intensity modulation. The number of beams will depend on the size of the prostate. In general, the number of beams increases with the size of the prostate. The standardization of the treatment plan will be done at the beam isocenter (prostate center of gravity). Leaves need to be adjusted so that the 85% isodose covers at least 95% of the PTV. The dose prescription will be done on the 85% isodose. The prescribed dose will be 6Gy per fraction. A heterogeneity correction will be mandatory for dosimetry calculation. The maximum point dose shall not, if possible, exceed 115% of the prescribed dose. Any dose greater than 115% of the prescribed dose shall not be outside of the prostate. This maximal dose shall be outside of the urethra. The dose at the urethra volume + 3 mm shall be, if possible, as close as possible to 6Gy and less than 6.5Gy. If not possible, more beams or intensity modulation can be used to decrease the dose at the urethra+3mm. An interval of 48 hours will be respected between each stereotactic radiotherapy session.

**Dose constraints**

| **Rectum** | **Bladder** | **Femoral heads** |
| --- | --- | --- |
| <75% of the prescribed dose in 20% of the volume | <75% of the prescribed dose in 20% of the volume | Maximum point <40 % of the prescribed dose |
| V65 < 30% | <55% of the prescribed dose in 35% of the volume with a maximum prescribed dose at 5% of the organ |
| <55% of the prescribed dose in 35% of the volume with a maximum prescribed dose at 5% of the organ |

### 7.3.2 By CyberKnife

#### Patient positioning

The patient will be in dorsal decubitus, in a stable position reproducible session after session. Patient immobilization must be sufficiently reliable in order to ensure that the Clinical Tumor Volume (CTV2) remains within the Planning Tumor Volume (PTV2).

#### Image acquisition

New image acquisition for planning purposes could be acquired at the discretion of the clinician according to the same guidelines described for phase 1.

#### Volume definitions

The prostate without the seminal vesicles will represent the Clinical Target Volume (CTV2). Prostate contouring will be done by a radiation oncologist experienced in the definition of prostatic volumes on scanner and MRI. MRI contours will be fused with the scanner contours in order to better define the prostatic apex.

The PTV2 will be obtained by an expansion of 5 mm around the CTV2.

The prostatic urethra will be delineated on MRI from the bladder up to 2 cm below the apex of prostate. A 3 mm margin will be defined around the urethra.

The rectum will be delineated with an internal expansion of 5 mm from 2 cm below the prostatic apex (without differentiation rectum / anal canal) up to the recto-sigmoid junction.

The empty bladder will be delineated in its totality with an internal expansion of 7 mm.

The femoral heads will be delineated up to the superior pole of the lesser trochanter

#### Control of prostate movements

The treatment is done using with CyberKnife “synchrony” module, to track the tumor lesion with the fiducials previously implanted. If tracking is not possible, the patient will be withdrawn from the study.

#### Localization of the prostate

Intra-prostatic markers will be implanted in the prostate before the first scanner acquisition of phase 1. The choice of markers will be left to the discretion of each participating center (gold seeds,…). They must ensure precise repositioning of the prostate (≤ 2 mm). They will also allow a precise fusion between the MRI and the planning scanner. At each stereotactic irradiation session, a precise repositioning (less or equal to 2 mm) of the prostate will be ensured.

#### Dosimetry

Beams will be produced by the CyberKnife (photon energy 6 MV). The number of beams shall be based on the prostate volume. The 85% isodose will cover 95% of the PTV. The prescribed dose will be of 6 Gy per fraction. A heterogeneity correction will be mandatory for dosimetry calculation. The maximum point dose shall not, if possible, exceed 115% of the prescribed dose. Any dose greater than 115% of the prescribed dose shall not be outside of the prostate. This maximal dose shall be outside of the urethra. An interval of 48 hours will be observed between two sessions of stereotactic radiotherapy.

**Dose constraints**

| **Rectum** | **Bladder** | **Femoral heads** |
| --- | --- | --- |
| <75% of the prescribed dose in 20% of the volume | <75% of the prescribed dose in 20% of the volume | Maximum point <40 % of the prescribed dose |
| V65 < 30% | <55% of the prescribed dose in 35% of the volume with a maximum prescribed dose at 5% of the organ |
| <55% of the prescribed dose in 35% of the volume with a maximum prescribed dose at 5% of the organ |

## 7.4 Independent Data Management Comittee (IDMC)

An independent Comittee will meet after the first ten patients and then on a regular basis (see chapter 8.5).

## 7.5 Subsequent antitumor treatments

In case of tumor progression, the patient will benefit from all treatments relevant to his situation. The patient presenting with distant progression remains in the study. Follow-up will be observed.

## 7.6 Concomitant treatments

**Before fiducials placement**

- In case of allergy or asthma : anti-allergy pre-medication will be prescribed
- Ticlid, Plavix, Previscan, Sintrom: treatment shall stop one week before placement
- Heparin, Calciparin, Fraxiparin: the last injection shall be given 12 hours before placement
- possible establishment of a heparin relay
- Aspirin: interruption for the preceding 10 days
- Glucophage : treatment will be interrupted 10 days before injection of contrast agent and restarted the day following creatinine check.

# 8 REGULATORY AND ETHICAL ASPECTS

## 8.1 Study management and sponsor's responsibilities (COL)

The study must be conducted in accordance with the ethical principles of the 1964 Helsinki declaration, revised in 2000 in Edinburgh, with the rules of Good Clinical Practice (GCP) defined by the International Conference on Harmonization (ICH-E6, 17/7/96) and with the provisions of the Public Health Act of August 9, 2004 and executive order of April 26, 2006.

The sponsor must archive all documents essential to study management, under safe conditions, for the minimum period defined by GCP, i.e. 15 years after completion of research.

## 8.2 Study management and investigator's responsibilities

The principal investigator for each centre concerned undertakes to manage the clinical trial in accordance with the protocol approved by the CPP and AFSSAPS. The investigator must not make any modifications to the protocol without the sponsor's authorization and without the CPP and AFSSAPS approving the proposed modifications.

The investigator is responsible:

- for providing the sponsor with his/her curriculum vitae, along with those of his/her co-investigators,
- for identifying the members of his/her team who are to take part in the trial and for defining their responsibilities,
- for initiating patient recruitment after receiving the sponsor's authorization,
- for making all necessary efforts to include the required number of patients, within the limits of the defined enrolment period.

Each investigator is responsible:

- for obtaining informed consent, personally dated and signed by the patient, prior to any trial-specific selection procedure,
- for regularly updating the case report forms (CRF) for each patient included in the trial and for providing the Clinical Research Associate (CRA) with direct access to the source documents to validate the CRF data,
- for dating, correcting and signing any CRF corrections for each patient included in the study,
- for welcoming regular visits from the CRA and, if applicable, those of auditors mandated by the sponsor, or by regulatory authority inspectors.

All documentation relative to the study (protocol, consent forms, CRF, investigator’s files, etc…) along with original documents (laboratory results, x-ray, consultation reports, clinical examinations reports, etc.) must be kept in a safe place and considered confidential.

The investigator is responsible for data archiving in accordance with current legislation. The latter must keep the data along with a patient identification list, for at least 15 years after the end of the study.

## 8.3 Committee for the Protection of Persons (CPP)

The clinical study protocol, along with its various amendments, is submitted by the study sponsor to a CPP in the region in which the co-coordinator practices his/her activity (art. L.1123-6).

## 8.4 Participant information and consent

Prior to performing biomedical research on an individual, the latter's **free, informative** and **explicit** consent must be obtained, after having been informed of the aims of the research, of the progress and duration of the study, of the potential study benefits, risks and requirements of the study, along with the type of product under study and the opinion given by the CPPs and AFSSAPS (art. L.1122-1).

The consent form must be personally dated and signed by the patient and investigator, or by the physician representing the patient (original filed by the investigator, a copy shall be issued to the patient or his/her legal representative) (appendix 8).

## 8.5 Independent monitoring committee

An independent monitoring committee for the trial will be established in order to guarantee protection of the patients, to ensure that the trial is conducted in an ethical fashion, to evaluate the risk/benefit ratio of the trial and to ensure the review of the scientific results during and at the end of the trial. The monitoring committee will meet 3 months after the inclusion of the ten fisrst patients and then on a regular basis.

Committe membership to be confirmed :

Two radiation oncologists : Professor STORME, Professor DUBRAY

An urologist : Professor VILLERS

A medical physicist : Doctor NOEL

- A methodologist : Mr KRAMAR

## 8.6 Confidentiality

In accordance with the Public Health Code, the investigators and all individuals required to collaborate in the study shall be held to professional secrecy concerning, in particular, the nature of the products used, the study itself, the test subjects and the results obtained. The investigator must ensure that his/her patients remain anonymous. The investigator shall keep a confidential patient identification list.

# 9 OPERATIONAL MANAGEMENT OF THE STUDY

## 9.1 Study organization

This study is sponsored by the Centre de Lutte Contre le Cancer du Nord de la France, Centre Oscar LAMBRET (COL).

## 9.2 Research costs and additional costs

Any additional costs stated in the Public Health Code are covered by an agreement negotiated between the COL and the centre representative, with consideration for the COL's financial means in the context of its sponsoring activity.

The COL shall, however, organize the study and shall provide the following materials (protocol, case report forms, investigator file) required for managing the study.

In case material or treatments are provided by other partners, the conditions must be specified in the study agreement.

## 9.3 Data collection and input - Study follow-up - Monitoring

Data are collected in a case report form (CRF) under the investigator's responsibility. These data are entered and validated in accordance with the study specifications. The Clinical Research Associate (CRA) assists the investigator in conducting the study. The CRA mandated by the sponsor makes a series of setup, follow-up and closure visits, in accordance with GCP.

## 9.4 Quality Assurance

The sponsor is responsible for implementing and maintaining a quality assurance system, as described in the COL procedures, in order to ensure that the study is conducted in accordance with the protocol and with GCP.

## 9.5 Data ownership and publication

At the end of the study, a report will be written by the sponsor, then validated by the principal investigators (Prof. E. LARTIGAU and Prof. O. CHAPET). No publication or presentation of the results of this trial will be done without the permission of the sponsor.

All publications will make reference at a minimum to :

- the Principal Investigators,
- all those having actively participated in the study. The order of co-authors will take into account the participation of the different trial investigators (number of patients included and evaluable).
- all those having contributed in a significant fashion to the progress of the study and publication process: The biostatistician, the manager of the Clinical Research Integrated Unit, and the sponsor.

In addition, any publication will include thanks to:

- CRA Sponsor,
- CRA Study Monitor,
- Technicians,
- Medical physicists,
- Research technicians in the participating centers,

The verification and agreement of the sponsor are required before any communication.

# 10 BIBLIOGRAPHY

1. Albertsen PC, Fryback DG, Storer BE, Kolon TF, Fine J. The impact of co-morbidity on life expectancy among men with localized prostate cancer. J Urol. 1996; 156(1): 127-32
2. Amer AM, Mott J, Mackay RI, Williams PC, Livsey J, Logue JP, Hendry JH. Prediction of the benefits from dose-escalated hypofractionated intensity-modulated radiotherapy for prostate cancer. Int J Radiat Oncol Biol Phys. 2003; 56(1): 199-207.
3. Ares C, Popowski M, Molla M, et al. Hypofractionated boost in Prostate cancer radiotherapy as part of two different dose escalation strategies, HDR brachytherapy or IMRT : a late rectal toxicity assessment. Int J Radiat Oncol Biol Phys 2007; 69: S477.
4. Beckendorf V, Guerif S, Le Prise E, Cosset JM, Lefloch O, Chauvet B, Salem N, Chapet O, Bourdin S, Bachaud JM, Maingon P, Lagrange JL, Malissard L, Simon JM, Pommier P, Hay MH, Dubray B, Luporsi E, Bey P. The GETUG 70 Gy vs. 80 Gy randomized trial for localized prostate cancer: feasibility and acute toxicity. Int J Radiat Oncol Biol Phys. 2004; 60(4): 1056-65.
5. Bentzen SM, Ritter MA The alpha beta ratio for prostate cancer: What is it, really ? Radiother Oncol 2005; 76: 1-3
6. Bey P, Beckendorf V, Aletti P, Marchesi V. Radiothérapie conformationnelle des cancers de la prostate : pour qui et comment ? Cancer Radiother. 2002; 6(3): 147-53.
7. Brenner DJ. Fractionation and late rectal toxicity. Int J Radiat Oncol Biol Phys. 2004; 60(4): 1013-5.
8. Brenner DJ, Martinez AA, Edmundson GK, Mitchell C, Thames HD, Armour EP. Direct evidence that prostate tumors show high sensitivity to fractionation (low alpha/beta ratio), similar to late-responding normal tissue. Int J Radiat Oncol Biol Phys. 2002; 52(1): 6-13.
9. Boike T, Anderson J, Novotny R, et al. Intra-fraction and inter-fraction prostate motion associated with stereotactic body radiation therapy (SBRT) and image guided radiation therapy (IGRT). Int J Radiat Oncol Biol Phys 2007; 69: S355.
10. Chirpaz E, Colonna M, Menegoz F, Grosclaude P, Schaffer P, Arveux P, Lesec'h JM, Exbrayat C, Schaerer R. Incidence and mortality trends for prostate cancer in 5 French areas from 1982 to 1996. Int J Cancer. 2002; 97(3): 372-6.
11. Dearnaley DP, Khoo VS, Norman AR, Meyer L, Nahum A, Tait D, Yarnold J, Horwich A. Comparison of radiation side-effects of conformal and conventional radiotherapy in prostate cancer: a randomised trial. Lancet. 1999; 353(9149): 267-72.
12. Demanes DJ, Rodriguez RR, Schour L, et al. High dose rate intensity modulated brachytherapy with external beam radiotherapy for prostate cancer : california endocurietherapy’s 10 years results. Int J Radiat Oncol Biol Phys 2005 ; 61 : 1306-16.
13. Dubray BM, Thames HD. Chronic radiation damage in the rat rectum: an analysis of the influences of fractionation, time and volume. Radiother Oncol. 1994; 33(1): 41-7.
14. Dubray B, Giraud P, Beaudre A. Glossaire de la radiothérapie conformationnelle. Cancer Radiother. 1999; 3(5): 360-6.
15. Eade TN, Hanlon AL, Horwitz EM, Buyyounouski MK, Hanks GE, Pollack A. [What dose of external-beam radiation is high enough for prostate cancer ?](../../../../pubmed/17398026%3Fordinalpos=12&itool=EntrezSystem2.PEntrez.Pubmed.Pubmed_ResultsPanel.Pubmed_DefaultReportPanel.Pubmed_RVDocSum) Int J Radiat Oncol Biol Phys. 2007 Jul 1; 68(3): 682-9.
16. Edwards M, Boike AT, Li T, Timmerman R. Relative motion of implanted prostate fiducials during stereotactic body radiation therapy. Int J Radiat Oncol Biol Phys 2007; 69(suppl): S376.
17. Eulau SM, Van Hollebeke L, Cavanagah W, et al. High dose rate iridium 192 brachytherapy in localized prostate cancer: results and toxicity with maximum follow up of 10 years. Int J Radiat Oncol Biol Phys 2000; 48: 525-33.
18. Fayers P, Aaronson N, Blordal K, Sullivan M. EORTC QLQ-C30 scoring manual. Bruxelles : EORTC,1995: 49.
19. Fédération Nationale des Centres de Lutte Contre le Cancer et Association Française d’Urologie. Standards, Options & Recommandations : cancer de la prostate non métastatique :John Libbey Eurotext Ed., Montrouge, 2002.
20. Fowler JF, Ritter MA, Chappell RJ, Brenner DJ. What hypofractionated protocols should be tested for prostate cancer? Int J Radiat Oncol Biol Phys. 2003; 56(4): 1093-104.
21. Fritz P, Kraus HJ, Muhlinickel W, et al. Stereotactic, single-dose irradiation of stage I non-small cell lung cancer and lung metastases. Radiat Oncol. 2006;20:30-8.
22. Galalae RM, Martinez AA, Mate T, et al. Long-term outcome by risk factors using conformal high dose rate brachytherapy (HDR-BT) boost with or without neoadjuvant androgen suppression for localized prostate cancer. Int J Radiat Oncol Biol Phys 2004; 58: 1048-55.
23. Hannoun-Levi JM, Ginot A, Thariat J ; L’antigène spécifique de prostate : modalités d’utilisation et interprétation. Cancer Radiother. 2008 ; 12(8) : 848-55.
24. Hara R, Itami J, Kondo T, et al. Clinical outcomes of single-fraction radiation therapy of lung tumors. Cancer 2006;106:1447-52.
25. Hoskin PJ, Motohashi K, Bownes P, Bryant L, Ostler P. High dose rate brachytherapy in combination with external beam radiotherapy in the radical treatment of prostate cancer: initial results of a randomised phase three trial. Radiother Oncol. 2007; 84(2): 114-20
26. Hoskin P. High dose rate brachytherapy for prostate cancer. Cancer Radiother. 2008 ; 12(6-7): 512-4
27. Junius S, Haustermans K, Bussels B, et al. [Hypofractionated intensity modulated irradiation for localized prostate cancer, results from a phase I/II feasibility study.](http://www.ncbi.nlm.nih.gov/pubmed/17686162?ordinalpos=1&itool=EntrezSystem2.PEntrez.Pubmed.Pubmed_ResultsPanel.Pubmed_DefaultReportPanel.Pubmed_RVDocSum) Radiat Oncol. 2007 ; 2: 29.
28. Kattan MW, Zelevsky MJ, Kupelian PA, Scardino PT, Fuks Z, Leibel SA : Pretreatment nomogram for predicting the outcome of three-dimensional conformal radiotherapy in prostate cancer. J Clin Oncol, 2000; 18: 3352-3359.
29. King CR, Brooks JD, Gill H, et al. Stereotactic body radiotherapy for localized prostate cancer: interim results of a prospective phase II clinical trial. Int J Radiat Oncol Biol Phys 2008 ; in press.
30. Koper PC, Jansen P, van Putten W, van Os M, Wijnmaalen AJ, Lebesque JV, Levendag PC. Gastro-intestinal and genito-urinary morbidity after 3D conformal radiotherapy of prostate cancer: observations of a randomized trial. Radiother Oncol. 2004; 73(1): 1-9.
31. Kupelian PA, Reddy CA, Carlson TP, Altsman KA, Willoughby TR. Preliminary observations on biochemical relapse-free survival rates after short-course intensity-modulated radiotherapy (70 Gy at 2.5 Gy/fraction) for localized prostate cancer. Int J Radiat Oncol Biol Phys. 2002; 53(4): 904-12.
32. Livsey JE, Cowan RA, Wylie JP, Swindell R, Read G, Khoo VS, Logue JP. Hypofractionated conformal radiotherapy in carcinoma of the prostate: five-year outcome analysis. Int J Radiat Oncol Biol Phys. 2003; 57(5): 1254-9.
33. Madsen BL, Hsi A, Pham HT, et al. Stereotactic hypofractionated accurate radiotherapy of the prostate (SHARP), 33.5 Gy in five fractions for localized disease: first clinical trial results. Int J Radiat Oncol Biol Phys 2007; 67: 1099-1105
34. Madsen BL, Hsi A, Pham HT, et al. Intrafractional stability of the prostate using a streotactic radiotherapy technique. Int J Radiat Oncol Biol Phys 2003; 57: 1285-91.
35. Mantz CA, Fernadez E, Harrison S et al. A phase II trial of trilogy based prostate SBRT : initial report of favorable acute toxicty outcomes. Int J Radiat Oncol Biol Phys 2007; 69 (suppl): S334.
36. Nyman J, Johansson KA, Hulten U. Stereotactic hypofractionated radiotherapy for stage I non small cell lung cancer_mature results for medically inoperable patients. Lung cancer 2006;51:97-103.
37. Partin AW, Mangold LA, Lamm DM, Walsh PC, Epstein JI, Pearson JD. Contemporary update of prostate cancer staging nomograms (Partin Tables) for the new millennium. Urology. 2001; 58(6): 843-8.
38. Remontet L, Esteve J, Bouvier AM, Grosclaude P, Launoy G, Menegoz F, Exbrayat C, Tretare B, Carli PM, Guizard AV, Troussard X, Bercelli P et al. Cancer incidence and mortality in France over the period 1978-2000. Rev Epidemiol Sante Publique. 2003; 51(1 Pt 1): 3-30.
39. Roach M, Hanks G, Thames H et al : Defining biochemical failure following radiotherapy with or without hormonal therapy in men with clinically localized prostate cancer : recommendations of the RTOG-ASTRO Phoenix consensus conference. Int. J. Rad. Oncol. Biol. Phys. 2006; 65(4): 965-974.
40. Rosen R C*, Riley A, Wagner G et al : The International Index of Erectile Function (IIEF) A Multidimen-tional scale for assessment of erectile dysfunction.Urology,1997; 49: 822
41. Rosen R.C, Cappelleri J C, Smith MD , Lipsky J and Pena BM Development and evaluation of an abridged, 5- item version of the international Index of Erectile Function IIEF-5 as a diagnostic tool for erectile dysfunction Inter-national Journal of Impotence Research 1999; 11: 319-326
42. Soulié M, Barré C, Beuzeboc P et al. Recommandations 2004 du CCAFU : Cancer de la Prostate. Progr. Urol. 2004 ; 14: 913-955
43. Svedman C, Karlsson K, Rutkowska E, et al. Stereotactic body radiotherapy of primary and metastatic renal lesions for patients with only one functioning kidney. Acta Oncol 2008;47:1578-83.
44. Thames HD Jr, Withers HR, Peters LJ, Fletcher GH. Changes in early and late radiation responses with altered dose fractionation: implications for dose-survival relationships. Int J Radiat Oncol Biol Phys. 1982; 8(2): 219-26.
45. Timmerman R, Mc Garry R, Yiannoutsos C et al. Excessive toxicity when treating central tumors in a phase II study of stereotactic body radiation therapy for medically inoperable earcly-stage lung cancer. J. Clin. Oncol. 2006; 24(30): 4833-39
46. Timmerman R, Papiez L, Mc Garry R, et al. Extracranial stereotactic results of a phase I study in medically inoperable stage I non-small cell lung cancer. Chest 2003;124(5):1946-55.
47. Tse RV, Hawkins M, Lockwood G, et al. Phase I of individualized stereotactic body radiotherapy for hepatocellular carcinoma and intrahepatic cholangiocarcinoma. J Clin Oncol 2008;26:657-64.
48. Xia T, Li H, Sun Q, et al. Promising clinical outcome of stereotactic body radiation therapy for patients with inoperable stage I/II non small cell lung cancer. Int J Radiat Oncol Biol Phys 2006;66:117-25.
49. Yeoh EE, Fraser RJ, McGowan RE, Botten RJ, Di Matteo AC, Roos DE, Penniment MG, Borg MF. Evidence for efficacy without increased toxicity of hypofractionated radiotherapy for prostate carcinoma: early results of a Phase III randomized trial. Int J Radiat Oncol Biol Phys. 2003; 55(4): 943-55.
50. Zimmermann FB, Geinitz H, Schill S et al. Stereotactic hypofractionated radiation therapy for stage I non-small cell lung cancer. Lung Cancer 2005; 48: 107-14.

# 11 APPENDICES

## Appendix 1: Study outline

Information / Consent

Inclusion

**1st phase**

**Conformal radiotherapy:**

**23 x 2 Gy**

Over 31 to 42 days (see chapter 7.2)

**2^nd^ phase**

**Stereotactic boost irradiation: 3 x 6 Gy**

Over 5 to 9 days(1)

CyberKnife or Linear Accelerator (see chapter 7.3)

D-21 D1 to D5 D8 to D12 D15 to D19 D22 to D26 D29 to 31 F 1 2 3

Start within 10 days of the last session of the 1^st^ phase

*Interval between fiducials placement and the planning CT-scan is 7 days*

Follow-up after the last stereotactic irradiation session : at 3, 6, 9 and 12 months

at 18, 24, 30 and 36 months (end of study)

then at 48 and 60 months

**in case of technical problem, the authorized postponement is 15 days*

## Appendix 2: Study timelines

|  | | Inclusion | | Fiducials | Immobilization | Dosi-metry | Conformal radiotherapy  31 to 42 days | Stereotactic boost irradiation  5 to 9 days | End of treatment  3 month | Monitoring  at, 6, 9, 12, 18, 24, 30 months | | Exit from the study at 36 months | | Monitoring  at 48 and 60 months | |
| --- | --- | --- | --- | --- | --- | --- | --- | --- | --- | --- | --- | --- | --- | --- | --- |
|  | **D-21** | |  | | | |  | **(6)** | **(4)** | **(4)** | **(4)** | | **(4)** | |  |
| **INFORMED CONSENT X** | | | | | | | | | | | | | | | |
| **EXAMINATIONS** | | | | | | | | | | | | | | | |
| Clinical examination, Weight, Height, PS, Rectal touch | | **X** | |  |  |  | **X (1)** | **X(7)** | **X** | **X** | | **X** | | **X** | |
| CBC, PLT, PT, TCA, INR | | **X** | |  |  |  |  |  |  |  | |  | |  | |
| Urea, creatinine, | | **X** | |  |  |  |  | **X(8)** | **X** |  | |  | |  | |
| PSA | | **X** | |  |  |  | **X(3)** | **X(8)** | **X** | **X** | | **X** | | **X** | |
| **TUMOR ASSESSMENT** | | | | | | | | | | | | | | | |
| Spiral CT (pelvis and prostate) | | **X** | |  |  |  |  |  |  |  | |  | |  | |
| Pelvis and prostate MRI | | **X** | |  |  |  |  |  | **X (5)** | **X (5)** | | **X (5)** | | **X (5)** | |
| Bone scan | | **X** | |  |  |  |  |  | **If clinically indicated** | | | | | | |
| **QUESTIONNARIES** | | | | | | | | | | | | | | | |
| IPSS | | **X** | |  |  |  |  |  | **X** | **X** | | **X** | | **X** | |
| IIEF-5 | | **X** | |  |  |  |  |  | **X** | **X** | | **X** | | **X** | |
| **TREATMENT PLANNING** | | | | | | | | | | | | | | | |
| Fiducial placement (recommended) | |  | | **X** |  |  |  |  |  |  | |  | |  | |
| CT scan (planning)(4) | |  | |  |  | **X (2)** |  |  |  |  | |  | |  | |
| Immobilization | |  | |  | **X** |  |  |  |  |  | |  | |  | |
| **TOXICITY** | | | | | | | | | | | | | | | |
| Adverse event | |  | |  |  |  | **X (1)** | **X(7)** | **X** | **X** | | **X** | | **X** | |

1. Weekly (except rectal touch)
2. 7 days interval between fiducial placement and acquisition of planning CT. For dosimetry, the most informative sequences of the planning CT Scan identified with the diagnostic CT-Scan fused with MRI
3. 48 hours after the last radiotherapy session
4. After the last radiotherapy session
5. Except if prior tumor progression
6. Start within the 10 days following the last irradiation session of the first phase
7. Before each radiotherapy session
8. Before stereotactic boost irradiation

## Appendix 3: Living conditions

|  | **Karnofsky scale** | | **WHO scale (1979)** | |
| --- | --- | --- | --- | --- |
| Physical ability, work ability | Intense, without difficulty | **100%** | **0** | Normal exterior activity |
|  | Normal + moderate discomfort | **90%** |  | No restriction |
|  | Reduced | **80%** | **1** | Reduction of intense efforts |
| Home activity | Normal, without assistance, but possible effort | **70%** | **2** | No exterior activity but ambulatory (50% of waking hours) |
|  | Requires occasional help with personal needs | **60%** | **3** | Strictly personal needs: bedridden > 50% of the time |
|  | Minimal + occasional assistance | **50%** |  |  |
| Incapable of feeding self | Permanent assistance | **40%** | **4** | Total incapacity |
|  | Frequently bedridden | **30%** |  | Frequently or constantly bedridden |
|  | Bedridden | **20%** |  |  |
|  | Moribund | **10%** |  |  |

## Appendix 4: TNM classification regarding prostate cancer

| **TUMOR** | | **LYMPH NODE** | | **METASTASIS** | |
| --- | --- | --- | --- | --- | --- |
| TX | Non-evaluable tumor | NX | Non-evaluable invasion | MX | Non-evaluable metastasis |
| T0 | No evidence of primary tumor | N0 | No nodal involvement | M0 | No metastases |
| **T1** | Tumor non-palpable or visible by imaging | N1 | Regional lymph node involvement | **M1** | **One (several) distant metastasis(es)** |
| T1a | Histologic evaluation: <5% of resected tissue |  |  | M1a | Non-regional lymph nodes |
| T1b | Histologic evaluation: >5% of resected tissue |  |  | M1b | Bone metastases |
| T1c | Discovered on biopsy |  |  | M1c | Other metastatic sites |
| **T2** | **Tumor confined with prostate** |  |  |  |  |
| T2a | Tumor limited to one lobe |  |  |  |  |
| T2b | Tumor involving the 2 lobes |  |  |  |  |
| **T3** | **Tumor extends through prostate capsule** |  |  |  |  |
| T3a | Extra-capsular extension |  |  |  |  |
| T3b | Tumor involving the seminal vesicles |  |  |  |  |
| **T4** | **Fixed tumor or invading adjacent structures (bladder neck, urethral sphincter, rectum, pelvic wall)** |  |  |  |  |

## Appendix 5: Gleason Score

**Gleason Score or GS =**
two numbers representing:
a) the predominant pattern and
b) the next most predominant pattern seen by the pathologist when examining prostate cancer tissue.

**Gleason Grade or GG =**
the primary grade is the most predominant pattern (“a” above). The secondary grade is the next most predominant pattern (“b” above).

**Gleason Differential or GD =**
the percentage makeup of the GS when there is any GG4 or GG5. This is shown as the GS followed by the GD, e.g. (4+3)[75/25]. In this example, GG4 comprised 75% of the primary grade.

## Appendix 6: Recommendations for planning CT-Scan before treatment

Date of planning CT-Scan

An interval of 7 days shall be respected between the placement of fiducial markers and the planning CT-scan.

Patient set-up

In treatment position

With possible immobilization system defined during treatment preparation

Requirements and scanner techniques

Spiral acquisition

Thickness of reconstructed slices 3 mm

Continuous acquisition

Tilt 0°

Maximum definition 512 x 512 x 512

Acquisition field

Total circumference of the patient

Including immobilization system

Injection

Upper and lower limits

According to the prescription

At minimum > 15 cm around the target volume and/or fiducial markers

## Appendix 7: marker specifications and placement recommendations for tracking

Fiducial marker specifications

Material Gold

Diameter 0.7 to 1.2 mm

Length 3 to 6 mm

Placement rules

Fasting, 5 hours before the examination

Verification of hemostasis, and possible correction

In case of allergy or asthma, an anti-allergic medication shall be prescribed

Concomitant treatment (see chapter 7.7.1)

Placement recommendations

During endo-rectal ultrasound

Minimal number of fiducial markers 3-4

Minimal space between two fiducial markers 1.5 cm

Minimum angle between two fiducial markers 15 °

Maximal distance from the irradiation target 0.5 mm

Maximal field comprising all fiducial markers 10 x 10 cm

Placement of fiducial markers shall precede planning CT- scan by at least 1 week

**1**

**4**

**2**

**1**

**3**

**4**

**2**

**3**

**Distance between fiducials : > 15 mm**

## Appendix 8: Informational letter to patient and consent form

*Trial title****:* intermediate risk prostate cancer : phase II prospective studY evaluating hypofractionated stereotactic boost irradiation**

Dear Sir,

The medical team caring for you is striving to improve all forms of treatment that may be available to you. A new treatment modality is proposed to you because it seems to be the local treatment that suits best your clinical situation.

In order to protect your healthy tissues (bladder and rectum), an optimal dose of radiation has to be administered with very high precision to your prostate cancer.

Recent progress in imaging and other technological breakthroughs have allowed treatment of prostatic lesions with “hypofractionated stereotactic irradiation”. Effectiveness of such radiation therapy has already been demonstrated in liver or brain lesions.

This « hypofractionated stereotactic irradiation” can be administered either by an upgraded linear accelerator or by CyberKnife (dedicated machine). This treatment is a boost of 3 fractions delivered after the end of the first treatment phase consisting of 23 fractions of 3D conformal radiation therapy. This boost will allow to increase the dose in your prostate thus a greater chance of cure while decreasing the dose to the nearby normal tissues.

The present document describes the study in which you are proposed to take part. It also summarizes the currently available information on the subject

1. **What is the aim of the study?**

The objective of this study is to evaluate the efficacy and tolerance of a new treatment modality: a stereotactic radiation therapy boost. A total of 76 patients will be enrolled in this study over a period of 2 years. The course of stereotactic radiation therapy treatment will consist of 3 sessions usually delivered over 8 to 10 week days following the end of the first 23 sessions of conventional radiation therapy.

1. **What are the study requirements?**

Before starting your treatment, an initial assessment is necessary. This will comprise complete questionnaire on your medical history, a physical examination, blood tests, a CT-scan, a bone scan and a MRI. You will be asked to complete a questionnaire assessing your quality of life.

Your prostate CT-scan of will be used to determine the location of the treatment zone and implant around the tumor, under local anesthesia, metal markers (size of a rice grain) called fiducials. Before the radiation therapy treatment, you will have a second CT-Scan to plan your treatment and mould your body in the immobilization mattress that will be used during all the irradiation sessions. This immobilization system is aimed to maintain your body, thus the zone to be treated, in the same position during each radiation session.

As soon as possible (about 2 to 3 weeks) after the first irradiation phase, the boost treatment will start and will consist of 3 irradiation sessions of approximately 2 hours each, over 8 to 10 days.

Just as before, your disease will be assessed with the same examinations as the ones performed initially every 3 months during 1 year then every 6 months during 4 years

If the results of the treatment are unsatisfactory, or if the treatment is not well tolerated and your physician decides to interrupt the treatment, or should you decide to withdraw from the study, your physician will then select the treatment best suited to your health condition.

Should you not wish to take part in this research, or should your participation in the research be interrupted for whatever reason, your follow-up and treatment will be provided by the same medical team, with no change in material conditions or in the quality of care.

3. What are the predictable risks?

The more frequent side-effects are:

- a rectum inflammation with an increase in frequency of stools,
- a bladder inflammation with more frequent urination

In all cases, specific treatments for these side effects will be administered with a view to reducing them.

If you so wish, you may, at any time, ask to be informed by your attending physician of the state of advancement of the research in which you are taking part and also of the results, once follow-up is complete.

**4. What are the expected benefits?**

Your participation in this study will give you the opportunity to increase your chance of cure by using a new treatment modality. This trial will also allow the scientists and the medical community to better know the benefits and the risks associated with this stereotactic radiation therapy for the treatment of your disease.

**5. What are your study participation rights and conditions?**

You may withdraw from the trial at any time, without justification and without any consequences on the remainder of your treatment, the quality of care, or on relations with your physician. You must notify the physician who proposed the study to you.

The sponsor of this trial has taken all steps provided by the law pertaining to the protection of persons submitting to biomedical research, Huriet act (no. 88-1138) of December 20, 1988, modified by the public health act (no. 2004-806) of August 9, 2004.

The sponsor has also, in accordance with current provisions, taken out an insurance policy with the Company SHAM *under no. ....* guaranteeing its civil liability, along with that of any person involved, as defined by article L 1121-7 of the Public Health Code.

The terms of this protocol have been submitted for approval to the Lille Nord-Ouest IV Committee for the Protection of Persons (CPP), whose mission is to verify that the conditions required for your protection have been met and your rights are respected. The Lille CPP issued a favorable opinion for the implementation of this protocol on .............

In relation with the biomedical research in which your physician is proposing you to participate, your personal data will be processed to analyze the results in light of the goal that has been presented to you. For this reason, the medical data relating to yourself, along with the study data, will be transmitted to the research Sponsor or to those individuals acting in its name in France. These data will be identified by a code number and your initials. These data may also, under conditions guaranteeing their confidentiality, be transmitted to French or foreign health authorities or to different Sponsor entities. In accordance with the provisions of the data protection act, you have a right to access and correct these data. You also have the right to oppose the transmission of the data covered by professional secrecy and likely to be used and processed in the context of this research. You may also access, either directly or through the intermediary of a physician of your choice, all of your medical data, by application of the provisions of article L 1111-7 of the Public Health Code. These rights are exercised with the physician following you in the context of the research and who knows your identity.

Furthermore, according to the provisions of the act of March 4, 2002 pertaining to patients' rights, you will be informed of the global trial results by the investigator, should you so wish

A copy of the patient information letter has been provided to you.

This protocol has been reviewed by the National Cancer League Patient Committee

**6. Who to contact should you have questions or problems?**

For any questions pertaining to your care in the context of this protocol and, in general terms of the study, your physician will be available at the following address in order to best serve your interests:

Dr ………………………………………….. Address:……………………………………….…………...…………………………………
Tel.: ……………………………………

**Biomedical research** **protocol** **consent form^(1)^**

1. *: all pages must be initialed by the investigator / co-investigator and the patient*

*Trial title****:* intermediate risk prostate cancer :**

**phase II prospective studY evaluating**

**hypofractionated stereotactic**

**boost irradiation**

**I, the undersigned :**

**Name : First name :**

**Address :**

have read and understood the information notice explaining the above-mentioned research protocol.

I have had the opportunity to ask any questions I had, I received the appropriate answers and was granted sufficient consideration time between the information and my decision to take part in this trial.

| Considering the information I have received :  *tick the boxes according to your intent (YES/NO)* | **YES** | **NO** |
| --- | --- | --- |
| **I accept, freely and willingly, to take part in the biomedical research ^(a, b)^**  N° IDRCB : …. |  |  |

*^(a)^ act no. 88-1138 of December 20, known as Huriet-Sérusclat act, pertaining to the protection of persons submitting to biomedical research, modified by public health act no. 2004-806 of August 9, 2004*

*^(b)^ act no. 2004-801 of August 6, 2004 pertaining to the protection of individuals with regard to the processing of data of personal nature, modifying act no. 78-17 of January 6, 1978 pertaining to data protection*

I have noted that I am free, at all times, to withdraw my participation. In that case, I will inform **Dr………………..**

I also understand that I have the right to oppose any automated processing of my personal data. Furthermore, possessing the right to access and correct the data pertaining to myself, I may exercise that right with **Dr………………..**

| _________________________________ | _________ | __________________ |
| --- | --- | --- |
| ***Patient’s or his legal representative’s name*** | ***Date*** | ***Signature*** |

| _________________________________ | ***_________*** | ***________________*** |
| --- | --- | --- |
| ***Investigator’s or physician representing him/her (co-investigator)’s name*** | ***Date*** | ***Signature*** |

1. *one co-signed copy must be issued to the person taking part in the research*

## Appendix 9: Declaration forms for serious adverse events

**NOTIFICATION OF A SERIOUS ADVERSE EVENT**

**To fax to the U.I.R.C. of the Centre Oscar LAMBRET at 03 20 29 59 71**

| **N° EudraCT/ID-RCB: Study code:** | | |
| --- | --- | --- |
| **❒ Serious Adverse Event EXPECTED** | | **❒ Serious Adverse Event UNEXPECTED** |
| **❒ Initial Report** | **❒ Follow-up report n°** | **Investigating Center n°** |

| 1. **PATIENT INFORMATION** | | | | | | | | | | |
| --- | --- | --- | --- | --- | --- | --- | --- | --- | --- | --- |
| Inclusion N°:  | Patient initials:   | | | | | | Date of birth:    | | | |
| Sex: ❒ F ❒ M | Weight (kg):  | | | Height (cm):  | | | Treatment arm: Lot N°:  | | | |
| 1. **INFORMATION REGARDING THE EVENT** | | | | | | | | | | |
| **Date of event occurrence:**     **Toxicity** (grade NCI-CTC V3.0): 1 ❒ 2 ❒ 3 ❒ 4 ❒ 5 ❒  **Adverse event:** ……………………………………………………………………………………………………..  **NCI – CTC v3.0 term:** ……………………………………………………………………………………………………………………………. | | | | | | | | | | |
| 1. **SEVERITY CRITERIA (check one or more boxes)** | | | | | | | | | | |
| ❒ Death, date  - Autopsy: ❒ Yes ❒ No  ❒ Disability / Temporary or permanent impairment ❒ Life threatening  ❒ Other cancer: ………………………………………….. ❒ Congenital anomaly or fetal malformation  ❒ Hospitalization (> 24 h), date ❒ Prolongation of hospitalization, date  | | | | | | | | | | |
| 1. **DESCRIPTION** | | | | | | | | | | |
|  | | | | | | | | | | |
| 1. **PROGRESSION OF THE EVENT TO DATE** | | | | | | | | | | |
| ❒ Continuing event ❒ Death related to event  ❒ Resolution without sequelae, date:    ❒ Death unrelated to event  ❒ Resolution with sequelae, date:    ❒ Unknown  Nature of sequelae: ……………………………………………………. Date of end of hospitalization:    | | | | | | | | | | |
| 1. **STUDY TREATMENTS** | | | | | | | | | | |
|  | |  | **Dates** | | | | | **Doses and Units** | | **Attributable** |
| Chemotherapy, radiotherapy,… | | Route | Treatment dates before onset of event | | | | | Last dose administered | Cumulative dose after first administration | 1 Excluded  2 Doubtful  3 Plausible  4 Probable  5 Very probable  6 Inconclusive |
| **1.** | |  |    | |    | | |  |  | **** |
| **2.** | |  |    | |    | | |  |  | **** |
| **3.** | |  |    | |    | | |  |  | **** |
| **4.** | |  |    | |    | | |  |  | **** |
| Medication administered: Was a code called? Yes ❒, result…………………….……………No ❒ , NA ❒ | | | | | | | | | | |
| Have one or more treatments been stopped?  ❒ Yes: N° 1 ❒ N° 2 ❒ N° 3 ❒ N° 4 ❒ N° 5 ❒, ❒ No ❒ NA  Resolution of event after stopping one or more products?  ❒ Yes: ❒ No ❒ NA | | | | | | Have one or more treatments been re-started?  ❒ Yes: N° 1 ❒ N° 2 ❒ N° 3 ❒ N° 4 ❒ N° 5 ❒, ❒ No ❒ NA  Reapparition of the event after re-introduction?  ❒ Yes: ❒ No ❒ NA | | | | |
| 1. **GLOBALLY ATTRIBUTABLE (in your opinion, this event is possibly related)** | | | | | | | | | | |
| ❒ to trial treatment(s) specify the name(s) of treatments ❒ to the progression of the disease  ❒ to the trial protocol ❒ other concomitant illness(es)  ❒ other concomitant treatment(s) ❒ other………………………………………………… | | | | | | | | | | |
| 1. **NOTIFIER** | | | | | | | | | | |
| Name and function of notifier: Date: Tel: Fax:  email: Signature of Investigator/Co-investigator: | | | | | | | | | | |

**If SERIOUS ADVERSE EVENT UNEXPECTED: ADDITIONAL INFORMATION**

**To fax to the U.I.R.C. of the Centre Oscar LAMBRET at 03 20 29 59 71**

| **N° EudraCT/ID-RCB: Study code: Inclusion N°:**  **Center N°:**  **Patient initials:**   |
| --- |

| **5. TREATMENTS** | | | | | | | | | | |
| --- | --- | --- | --- | --- | --- | --- | --- | --- | --- | --- |
|  | **Route** | **Dates** | | **Doses and Units** | | | **Treatment modifications** | **Treatments** | | |
|  |  | Treatment dates before onset of event | | Last dose | Cumulative dose after first administration | | 1: dose reduction  2: temporary interruption  3: permanent interruption | Resolution of AE after stopping treatment  1: Yes  2: No  3: NA | Reapparition of AE after  re-introduction  1: Yes  2: No  3: NA | Attributable* |
| …………………………………… |  | From     | To     |  |  | |      Date |      Date |      Dose:  |  |
| …………………………………… |  | From     | To     |  |  | |      Date |      Date |      Dose:  |  |
| …………………………………… |  | From     | To     |  |  | |      Date |      Date |      Dose:  |  |
| …………………………………… |  | From     | To     |  |  | |      Date |      Date |      Dose:  |  |
| …………………………………… |  | From     | To     |  |  | |      Date |      Date |      Dose:  |  |
| …………………………………… |  | From     | To     |  |  | |      Date |      Date |      Dose:  |  |
| …………………………………… |  | From     | To     |  | |  |      Date |      Date |      Dose:  |  |

*attributable: 1 = excluded, 2 = doubtful, 3 = plausible, 4 = probable, 5 = very probable, 6 = inconclusive

## Appendix 10 : NCI-CTCAE Version 3.0 Criteria

| **Quick Reference**  The NCI Common Terminology Criteria for Adverse Events v3.0 is a descriptive terminology which can be utilized for Adverse Event (AE) reporting. A grading (severity) scale is provided for each AE term. | **REMARK** A 'REMARK' is a clarification of an AE. **ALSO CONSIDER** An 'ALSO CONSIDER' indicates additional AEs that are to be graded if they are clinically significant  **NAVIGATION NOTE** A 'NAVIGATION NOTE' indicates the location of an AE term within the CTCAE document. It lists signs/symptoms alphabetically and the CTCAE term will appear in the same CATEGORY unless the 'NAVIGATION NOTE' States differently |
| --- | --- |

|  | | | GRADE | | | | | | | | | | |
| --- | --- | --- | --- | --- | --- | --- | --- | --- | --- | --- | --- | --- | --- |
| **Adverse Event** | **Short Name** | | **1** | | **2** | | **3** | | **4** | | **5** | | |
| **AUDITORY / EAR** | | | | | | | | | | | | | |
| Tinnitus | Tinnitus | | | — | Tinnitus not interfering with ADL | | Tinnitus interfering with ADL | | Disabling | |  | | |
| Auditory/Ear – Other (Specify ) | Auditory/Ear – Other (Specify) | | | Mild | Moderate | | Severe | | Life-threatening; disabling | | Death | | |
| **BLOOD/ BONE MARROW** | | | | | | | | | | | | | |
| Bone marrow cellularity | Bone marrow cellularity | | | Mildly hypocellular or ≤25% reduction from normal cellularity for age | Moderately hypocellular or >25 – ≤50% reduction from normal cellularity for age | | Severely hypocellular or >50 – ≤75% reduction cellularity from normal for age | | — | | Death | | |
| Hemoglobin | Hemoglobin | | | <LLN – 10.0 g/dL  <LLN – 6.2 mmol/L  <LLN – 100 g/L | <10.0 – 8.0 g/dL <6.2 – 4.9 mmol/L <100 – 80g/L | | <8.0 – 6.5 gIdL <4.9 – 4.0 mmol/L <80 – 65 g/L | | <6.5 g/dL <4.0 mmol/L <65 g/L | | Death | | |
| Leukocytes (total WBC) | Leukocytes | | | <LLN – 3000/mm^3^ <LLN -3.0x10**^9^**/L | <3000–2000/mm^3^ <3.0–2.0x10^9^/L | | <2000–1000/mm**^3^** <2.0–1.0x10**^9^**/L | | <1000/mm^3^ <1.0x10^9^/L | | Death | | |
| Lymphopenia | Lymphopenia | | | <LLN – 800/mm^3^ <LLN x0.8–10^9^/L | <800– 500/mm^3^ <0.8–0.5x10^9^/L | | <500 – 200 mm^3^ <0.5–0.2x10**^9^**/L | | <200/mm**^3^** <0.2x10**^9^**/L | | Death | | |
| Neutrophils/Granulocytes (ANC/AGC) | Neutrophils | | | <LLN – 1500/mm**^3^** <LLN -1.5 x 10^9^ /L | <1500– 1000/mm^3^ <1.5 – 1.0 x 10^9^ /L | | <1000 – 500/mm^3^ <1.0 – 0.5 x 10^9^ /L | | <500/mm^3^ <0.5 x 10^9^ /L | | Death | | |
| Platelets | Platelets | | | <LLN –75,000/mm^3^ <LLN–75 0 x 10^9^ /L | <75,000 –50,000/mm^3^ <75.0–50.0x10^9^ /L | | <50,000 – 25,000/mm^3^ <50.0 – 25.0x10^9^/L | | <25,000/mm**^3^** <25.0 x 10^9^ /L | | Death | | |
| Blood/Bone Marrow – Other (Specify) | Blood – Other (Specify) | | | Mild | Moderate | | Severe | | Life-threatening; disabling | | Death | | |
| **CARDIAC ARRHYTHMIA** | | | | | | | | | | | | | |
| Palpitations | Palpitations | | | Present | Present with associated symptoms (e.g., lightheadedness, shortness of breath) | | — | | — | | — | | |
| REMARK: Grade palpitations only in the absence of a documented arrhythmia. | | | | | | | | | | | | | |
| Supraventricular and nodal arrhythmia | Supraventricular arrhythmia – | | | Asymptomatic, intervention not indicated | Non-urgent medical intervention indicated | | Symptomatic and incompletely controlled medically, or controlled with device (e.g., pacemaker) | | Life-threatening (e.g., arrhythmia associated with CHF, hypotension, syncope, shock) | | Death | | |
| – Sinus tachycardia |  | | |  |  | |  | |  | |  | | |
| Vasovagal episode | Vasovagal episode | | | — | Present without loss of consciousness | | Present with loss of consciousness | | Life-threatening consequences | | Death | | |
| Cardiac Arrhythmia – Other (Specify,) | Cardiac Arrhythmia –Other (Specify) | | | Mild | Moderate | | Severe | | Life-threatening; disabling | | Death | | |
| **CARDIAC GENERAL** | | | | | | | | | | | | | |
| Cardiac ischemia/infarction | Cardiac ischemia/infarction | | Asymptomatic arterial narrowing without ischemia | | Asymptomatic and testing suggesting ischemia; stable angina | | Symptomatic and testing consistent with ischemia; unstable angina; intervention indicated | | Acute myocardial infarction | | Death | | |
| Cardiopulmonary arrest, cause unknown (non-fatal) | Cardiopulmonary arrest | | — | | — | | — | | Life-threatening | | — | | |
| REMARK: Grade 4 (non-fatal) is the only appropriate grade. CTCAE provides three alternatives for reporting Death: | | | | | | | | | | | | | |
| 1. A CTCAE term associated with Grade 5. 2. A CTCAE 'Other (Specify)’, within any CATEGORY. 3. Death not associated with CTCAE term – *Select* in the DEATH CATEGORY. | | | | | | | | | | | | | |
| Hypertension | Hypertension | | | Asymptomatic, transient (<24 hrs) increase by >20 mmHg (diastolic) or to >150/100 if previously WNL; intervention not indicated | Recurrent or persistent (>24 hrs) or symptomatic increase by >20 mmHg (diastolic) or to >150/100 if previously WNL; monotherapy may be indicated | | Requiring more than one drug or more intensive therapy than previously | | Life-threatening consequences (e.g., hypertensive crisis) | | Death | | |
| Hypotension | Hypotension | | | Changes, intervention not indicated | Brief (<24 hrs) fluid replacement or other therapy; no physiologic consequences | | Sustained (>24 hrs) therapy, resolves without persisting physiologic consequences | | Shock (e.g., acidemia; impairment of vital organ function) | | Death | | |
| Also CONSIDER: Syncope (fainting). | | | | | | | | | | | | | |
| Left ventricular systolic dysfunction | Left ventricular systolic dysfunction | | | Asymptomatic, resting ejection fraction (EF) <60 – 50%; shortening fraction (SF) <30 – 24% | Asymptomatic, resting EF <50 – 40%; SF <24 – 15% | | Symptomatic CHF responsive to intervention; EF <40 – 20% SF <15% | | Refractory CHF or poorly controlled; EF <20%; intervention such as ventricular assist device, ventricular reduction surgery, or heart transplant indicated | | Death | | |
| Pericardial effusion (non-malignant) | Pericardial effusion | | | Asymptomatic effusion | — | | Effusion with physiologic consequences | | Life-threatening consequences (e.g., tamponade); emergency intervention indicated | | Death | | |
| Pericarditis | Pericarditis | | | Asymptomatic, ECG or physical exam (rub) changes consistent with pericarditis | Symptomatic pericarditis (e.g., chest pain) | | Pericarditis with physiologic consequences (e.g., pericardial constriction) | | Life-threatening consequences; emergency intervention indicated | | Death | | |
| Cardiac General – Other (Specify) | Cardiac General – Other (Specify) | | | Mild | Moderate | | Severe | | Life-threatening; disabling | | Death | | |
| **CONSTITUTIONAL SYMPTOMS** | | | | | | | | | | | | | |
| Fatigue (asthenia, lethargy, malaise) | Fatigue | | | Mild fatigue over baseline | Moderate or causing difficulty performing some ADL | | Severe fatigue interfering with ADL | | Disabling | | — | | |
| Fever (in the absence of neutropenia, where neutropenia is defined as ANC <1.0 x 10^9^/L) | Fever | | | 38.0 — 39.0°C | >39.0 — 40.0°C | | >40.0°C for <24 hrs | | >40.0°C for >24 hrs | | Death | | |
| REMARK: The temperature measurements listed are oral or tympanic.  ALSO CONSIDER: Allergic reaction/hypersensitivity(including drug fever). | | | | | | | | | | | | | |
| Insomnia | Insomnia | | | Occasional difficulty sleeping, not interfering with function | Difficulty sleeping, interfering with function but not interfering with ADL | | Frequent difficulty sleeping, interfering with ADL | | Disabling | | — | | |
| REMARK: If pain or other symptoms interfere with sleep, do NOT grade as insomnia. Grade primary event(s) causing insomnia. | | | | | | | | | | | | | |
| Sweating (diaphoresis) | Sweating | | | Mild and occasional | Frequent or drenching | | — | | — | | — | | |
| ALSO CONSIDER: Hot flashes/flushes. | | | | | | | | | | | | | |
| Weight gain | Weight gain | | | 5 – <10% of baseline | 10 – <20% of baseline | | ≥ 20% of baseline | | — | | — | | |
| REMARK: Edema, depending on etiology, is graded in the CARDIAC GENERAL or LYMPHATICS CATEGORIES.  ALSO CONSIDER: Ascites (non-malignant); Pleural effusion (non-malignant). | | | | | | | | | | | | | |
| Weight loss | Weight loss | | | 5 to <10% from baseline; intervention not indicated | 10 – <20% from baseline; nutritional support indicated | | ≥20% from baseline; tube feeding or TPN indicated | | — | | — | | |
| Constitutional Symptoms – Other (Specify) | Constitutional Symptoms – Other (Specify) | | | Mild | Moderate | | Severe | | Life-threatening; disabling | | Death | | |
| **DERMATOLOGY/SKIN** | | | | | | | | | | | | | |
| Cheilitis | Cheilitis | | | Asymptomatic | Symptomatic, not interfering with ADL | | Symptomatic, interfering with ADL | | — | | — | | |
| Dry skin | Dry skin | | | Asymptomatic | Symptomatic, not interfering with ADL | | Interfering with ADL | | — | | — | | |
| Flushing | Flushing | | | Asymptomatic | Symptomatic | | — | | — | | — | | |
| Hair Ioss/alopecia (scalp or body) | Alopecia | | | Thinning or patchy | Complete | | — | | — | | — | | |
| Hyperpigmentation | Hyperpigmentation | | | Slight or Localized | Marked or generalized | | — | | — | | — | | |
| Hypopigmentation | Hypopigmentation | | | Slight or localized | Marked or generalized | | — | | — | | — | | |
| Induration/fibrosis (skin and subcutaneous tissue) | Induration | | | Increased density on palpation | Moderate impairment of function not interfering with ADL; marked increase in density and firmness on palpation with *or* without minimal retraction | | Dysfunction interfering with ADL; very marked density, retraction or fixation | | — | | — | | |
| Also CONSIDER: Fibrosis-cosmesis; Fibrosis-deep connective tissue. | | | | | | | | | | | | | |
| Nail changes | Nail changes | | | Discoloration; ridging (koilonychias); pitting | Partial or complete loss of nail(s); pain in nailbed(s) | | Interfering with ADL | | — | | — | | |
| NAVIGATION NOTE: Petechiae is graded as Petechiae/purpura (hemorrhage/bleeding into skin or mucosa) in the HEMORRHAGE/BLEEDING CATEGORY | | | | | | | | | | | | | |
| Photosensitivity | Photosensitivity | | | Painless erythema | Painful erythema | | Erythema with desquamation | | Life-threatening; disabling | | Death | | |
| Pruritus/itching | Pruritus | | | Mild or localized | Intense or widespread | | Intense or widespread and interfering with ADL | | — | | — | | |
| ALSO CONSIDER: Rash/desquamation. | | | | | | | | | | | | | |
| Rash/ desquamation | Rash | | | Macular or papular eruption or erythema without associated symptoms | Macular or papular eruption or erythema with pruritus or other associated symptoms; localized desquamation or other lesions covering <50% of body surface area (BSA) | | Severe, generalized erythroderma or macular, papular or vesicular eruption; desquamation covering ≥50% BSA | | Generalized exfoliative, ulcerative, or bullous dermatitis | | Death | | |
| REMARK: Rash/desquamation may be used for GVHD. | | | | | | | | | | | | | |
| Rash: acne/acneiform | Acne | | | Intervention not indicated | Intervention indicated | | Associated with pain, disfigurement, ulceration, or desquamation | | — | | Death | | |
| Rash: erythema multiforme (e.g., Stevens-Johnson syndrome, toxic epidermal necrolysis) | Erythema multiforme | | | — | Scattered, but not generalized eruption | | Severe (e.g., generalized rash or painful stomatitis); IV fluids, tube feedings, or TPN indicated | | Life-threatening; disabling | | Death | | |
| Urticaria (hives, welts, wheals) | Urticaria | | | Intervention not indicated | Intervention indicated for <24 hrs | | Intervention indicated for ≥24 hrs | | — | | — | | |
| ALSO CONSIDER: Allergic reaction/hypersensitivity (including drug fever). | | | | | | | | | | | | | |
| Dermatology/Skin – Other | Dermatology – Other Mild | | | Moderate | Severe | | Life-threatening; disabling | | Death | |  | | |
| **ENDOCRINE** | | | | | | | | | | | | | |
| Hot flashes/flushes | Hot flashes | | | Mild | Moderate | | Interfering with ADL | | — | | — | | |
| Endocrine - Other  (Specify’, _) | Endocrine – Other (Specify) | | | Mild | Moderate | | Severe | | Life-threatening; disabling | | Death | | |
|  | | | | | | | | | | | | | |
| **GASTROINTESTINAL** | | | | | | | | | | | | | |
| NAVIGATION NOTE: Abdominal pain or cramping is graded as Pain – *Select* in the PAIN CATEGORY. | | | | | | | | | | | | | |
| Anorexia | Anorexia | | | Loss of appetite without alteration in eating habits | Oral intake altered without significant weight loss or malnutrition; oral nutritional supplements indicated | | Associated with significant weight loss or malnutrition (e.g., inadequate oral caloric and/or fluid intake); IV fluids, tube feedings or TPN indicated | | Life-threatening consequences | | Death | | |
| ALSO CONSIDER: Weight loss. | | | | | | | | | | | | | |
| Ascites (non-malignant) | Ascites | | | Asymptomatic | Symptomatic, medical intervention indicated | | Symptomatic, invasive procedure indicated | | Life-threatening consequences | | Death | | |
| REMARK: Ascites (non-malignant) refers to documented non-malignant ascites or unknown etiology, but unlikely malignant, and includes chylous ascites. | | | | | | | | | | | | | |
| Colitis | Colitis | | | Asymptomatic, pathologic or radiographic findings only | Abdominal pain; mucus or blood in stool | | Abdominal pain, fever, change in bowel habits with ileus; peritoneal signs | | Life-threatening consequences (e.g., perforation, bleeding, ischemia, necrosis, toxic megacolon) | | Death | | |
| ALSO CONSIDER: Hemorrhage, GI – *Select.* | | | | | | | | | | | | | |
| Constipation | Constipation | | | Occasional or intermittent symptoms; occasional use of stool softeners, laxatives, dietary modification, or enema | Persistent symptoms with regular use of laxatives or enemas indicated | | Symptoms interfering with ADL; constipation with manual evacuation indicated | | Life-threatening consequences (e.g., obstruction, toxic megacolon) | | Death | | |
| ALSO CONSIDER: Ilieus, GI (functional obstruction of bowel, i.e., neuroconstipation); Obstruction, GI – *Select.* | | | | | | | | | | | | | |
| Dehydration | Dehydration | | | Increased oral fluids indicated; dry mucous membranes; diminished skin turgor | IV fluids indicated <24 hrs | | IV fluids indicated ≥24 hrs | | Life-threatening consequences (e.g., hemodynamic collapse) | | Death | | |
| ALSO CONSIDER: Diarrhea; Hypotension; Vomiting | | | | | | | | | | | | | |
| Diarrhea | Diarrhea | | | Increase of <4 stools per day over baseline; mild increase in ostomy output compared to baseline | Increase of 4 - 6 stools per day over baseline; IV fluids indicated <24hrs; moderate increase in ostomy output compared to baseline; not interfering with ADL | | Increase of ≥7 stools per day over baseline; incontinence; IV fluids ≥24 hrs; hospitalization; severe increase in ostomy output compared to baseline; interfering with ADL | | Life-threatening consequences (e.g., hemodynamic collapse) | | Death | | |
| REMARK: Diarrhea includes diarrhea of small bowel or colonic origin, and/or ostomy diarrhea. Also CONSIDER: Dehydration; Hypotension. | | | | | | | | | | | | | |
| Distension/bloating, abdominal | Distension | | | Asymptomatic | Symptomatic, but not interfering with GI function | | Symptomatic, interfering with GI function | | — | | — | | |
| Also CONSIDER: Ascites (non-malignant); Ilieus, GI (functional obstruction of bowel, i.e., neuroconstipation); Obstruction, GI – *Select.* | | | | | | | | | | | | | |
| Dry mouth/salivary gland (xerostomia) | Dry mouth | | | Symptomatic (dry or thick saliva) without significant dietary alteration; unstimulated saliva flow >0.2 ml/min | Symptomatic and significant oral intake alteration (e.g., copious water, other lubricants, diet limited to purees and/or soft, moist foods); unstimulated saliva 0.1 to 0.2 ml/min | | Symptoms leading to inability to adequately aliment orally; IV fluids, tube feedings, or TPN indicated; unstimulated saliva <0.1 ml/min | | — | | — | | |
| REMARK: Dry mouth/salivary gland (xerostomia) includes descriptions of grade using both subjective and objective assessment parameters. Record this event consistently throughout a patient's participation on study. If salivary flow measurements are used for initial assessment, subsequent assessments must use salivary flow. | | | | | | | | | | | | | |
| ALSO CONSIDER: Salivary gland changes/saliva. | | | | | | | | | | | | | |
| Flatulence | Flatulence | | | Mild | Moderate | | — | | — | | — | | |
| Gastritis (including bile reflux gastritis) | Gastritis | | | Asymptomatic radiographic or endoscopic findings only | Symptomatic; altered gastric function (e.g., inadequate oral caloric or fluid intake); IV fluids indicated <24 hrs | | Symptomatic and severely altered gastric function (e.g., inadequate oral caloric or fluid intake); IV fluids, tube feedings, or TPN indicated ≥24 hrs | | Life-threatening consequences; operative intervention requiring complete organ resection (e.g., gastrectomy) | | Death | | |
| Also CONSIDER: Hemorrhage, GI – Select, Ulcer, GI – *Select.* | | | | | | | | | | | | | |
| Heartburn/ dyspepsia | Heartburn | | | Mild | Moderate | | Severe | | — | | — | | |
| lleus, GI (functional obstruction of bowel, i.e., neuroconstipation) | lleus | | | Asymptomatic, radiographic findings only | Symptomatic; altered GI function (e.g., altered dietary habits); IV fluids indicated <24 hrs | | Symptomatic and severely altered GI function; IV fluids, tube feeding, or TPN indicated ≥24 hrs | | Life-threatening consequences | | Death | | |
| REMARK: Ilieus, GI is to be used for altered upper or lower Gl function (e.g., delayed gastric or colonic emptying). | | | | | | | | | | | | | |
| Also CONSIDER: Constipation; Nausea; Obstruction, GI – *Select,* Vomiting. | | | | | | | | | | | | | |
| Mucositis/stomatitis (clinical exam)  — *Select*  *—* Anus — Esophagus — Large bowel — Larynx — Oral cavity — Pharynx — Rectum — Small bowel — Stomach — Trachea | Mucositis (clinical exam) — *Select* | | | Erythema of the mucosa | Patchy ulcerations or pseudomembranes | | Confluent ulcerations or pseudomembranes; bleeding with minor trauma | | Tissue necrosis; significant spontaneous bleeding; life-threatening consequences | | Death | | |
| REMARK: Mucositis/stomatitis (functional/symptomatic) may be used for mucositis of the upper aero-digestive tract caused by radiation, agents, or GVHD. | | | | | | | | | | | | | |
| Mucositis/stomatitis (functional/symptomatic)  — *Select:*  *—* Anus — Esophagus — Large bowel — Larynx — Oral cavity — Pharynx — Rectum — Small bowel — Stomach — Trachea | Mucositis (functional/ symptomatic) — *Select* | | | Upper aerodiqestive tract  sites: Minimal symptoms,normal diet; minimal respiratory symptoms but not interfering with function  Lower Gl sites:  Minimal discomfort, intervention not indicated | Upper aerodiqestive tract  sites: Symptomatic but can eat and swallow modified diet; respiratory symptoms interfering with function but not interfering with ADL  Lower Gl sites:  Symptomatic, medical intervention indicated but not interfering with ADL | | Upper aerodiqestive tract ites:  Symptomatic and unable to adequately aliment or hydrate orally; respiratory symptoms interfering with ADL  Lower Gl sites:  Stool incontinence or other symptoms interfering with ADL | | Symptoms associated with life-threatening consequences | | Death | | |
| Nausea | Nausea Vomiting. | | | Loss of appetite without alteration in eating habits | Oral intake decreased without significant weight loss, dehydration or malnutrition; IV fluids indicated <24 hrs | | Inadequate oral caloric or fluid intake; IV fluids, tube feedings, or TPN indicated ≥24 hrs | | Life-threatening consequences | | Death | | |
| Also CONSIDER: Anorexia; | | | | | | | | | | | | | |
| Obstruction, GI – *Select*  – Cecum – Colon – Duodenum – Esophagus – Gallbladder – Ileum – Jejunum – Rectum – Small bowel NOS – Stoma – Stomach | Obstruction, GI – Select | | | Asymptomatic radiographic findings only | Symptomatic; altered GI function (e.g., altered dietary habits, vomiting, diarrhea, or GI fluid loss); IV fluids indicated <24 hrs | | Symptomatic and severely altered GI function (e.g., altered dietary habits, vomiting, diarrhea, or GI fluid loss); IV fluids, tube feedings, or TPN indicated ≥24 hrs; operative intervention indicated | | Life-threatening consequences; operative intervention requiring complete organ resection (e.g., total colectomy) | | Death | | |
| Taste alteration (dysgeusia) | Taste alteration | | | Altered taste but no change in diet | Altered taste with change in diet (e.g., oral supplements); noxious or unpleasant taste; Ioss of taste | | — | | — | | — | | |
| Ulcer, GI – Select. – Anus – Cecum – Colon – Duodenum – Esophagus – Ileum – Jejunum – Rectum – Small bowel NOS – Stoma – Stomach | Ulcer, GI – *Select* | | | Asymptomatic, radiographic or endoscopic findings only | Symptomatic; altered GI function (e.g., altered dietary habits, oral supplements); IV fluids indicated <24 hrs | | Symptomatic and severely altered GI function (e.g., inadequate oral caloric or fluid intake); IV fluids, tube feedings, or TPN indicated ≥24 hrs | | Life-threatening consequences | | Death | | |
| ALSO Consider: Hemorrhage, GI – *Select. Ulcer* | | | | | | | | | | | | | |
| Vomiting | Vomiting | | | 1 episode in 24 hrs | 2 – 5 episodes in 24 hrs; IV fluids indicated <24 hrs | | ≥6 episodes in 24 hrs; IV fluids, or TPN indicated ≥24 hrs | | Life-threatening consequences | | Death | | |
| Also CONSIDER: Dehydration. | | | | | | | | | | | | | |
| Gastrointestinal – Other (Specify,) | GI – Other (Specify) | | | Mild | Moderate | | Severe | | Life-threatening; disabling | | Death | | |
| **HEMORRHAGE/BLEEDING** | | | | | | | | | | | | | |
| Hematoma | Hematoma | | | Minimal symptoms, invasive intervention not indicated | Minimally invasive evacuation or aspiration indicated | | Transfusion, interventional radiology, or operative intervention indicated | | Life-threatening consequences; major urgent intervention indicated | | Death | | |
| REMARK: Hematoma refers to extravasation at wound or operative site or secondary to other intervention. Transfusion implies pRBC. Also CONSIDER: Fibrinogen; INR (International Normalized Ratio of prothrombin time); Platelets; PTT (Partial Thromboplastin Time). | | | | | | | | | | | | | |
| Hemorrhage, CNS | CNS hemorrhage | | | Asymptomatic, radiographic findings only | Medical intervention indicated | | Ventriculostomy, ICP monitoring, intraventricular thrombolysis, or operative intervention indicated | | Life-threatening consequences; neurologic deficit or disability | | Death | | |
| ALso CONSIDER: Fibrinogen; INR (International Normalized Ratio of prothrombin time); Platelets; PTT (Partial Thromboplastin Time). | | | | | | | | | | | | | |
| Hemorrhage, GI – *Select. –* Abdomen NOS – Anus – Biliary tree – Cecum/appendix – Colon – Duodenum – Esophagus – Ileum – Jejunum – Liver – Lower GI NOS – Oral cavity – Pancreas – Peritoneal cavity – Rectum – Stoma – Stomach – Upper GI NOS – Varices (esophageal) – Varices (rectal) | Hemorrhage, GI – Select | | | Mild, intervention (other than iron supplements) not indicated | Symptomatic and medical intervention or minor cauterization indicated | | Transfusion, interventional radiology, endoscopic, or operative intervention indicated; radiation therapy (i.e., hemostasis of bleeding site) | | Life-threatening consequences; major urgent intervention indicated | | Death | | |
| REMARK: Transfusion implies pRBC. | | | | | | | | | | | | | |
| ALSO CONSIDER: Fibrinogen; INR (International Normalized Ratio of prothrombin time); Platelets; PTT (Partial Thromboplastin Time). | | | | | | | | | | | | | |
| Hemorrhage, pulmonary/ upper respiratory  – Nose | Hemorrhage pulmonary – *Select* | | | Mild, intervention not indicated | Symptomatic and medical intervention indicated | | Transfusion, interventional radiology, endoscopic, or operative intervention indicated; radiation therapy (i.e., hemostasis of bleeding site) | | Life-threatening consequences; major urgent intervention indicated | | Death | | |
| REMARK: Transfusion implies pRBC. Also CONSIDER: Fibrinogen; INR (International Normalized Ratio of prothrombin time); Platelets; PTT (Partial Thromboplastin Time). | | | | | | | | | | | | | |
| Petechiae/purpura (hemorrhage/bleeding into skin or mucosa) | Petechiae | | Few petechiae | | Moderate petechiae; purpura | | Generalized petechiae or purpura | | — | | — | | |
| ALSO CONSIDER: Fibrinogen; INR (International Normalized Ratio of prothrombin time); Platelets; PTT (Partial Thromboplastin Time). | | | | | | | | | | | | | |
| NAVIGATION NOTE: Vitreous hemorrhage is graded in the OCULAR/VISUAL CATEGORY | | | | | | | | | | | | | |
| Hemorrhage/ Bleeding – Other (Specify,_) | Hemorrhage – Other (Specify,_) | | Mild without transfusion | | — | | Transfusion indicated | | Catastrophic bleeding, requiring major non-elective intervention | | Death | | |
| **HEPATOBILIARY/PANCREAS** | | | | | | | | | | | | | |
| Liver dysfunction/failure (clinical) | | Liver dysfunction | — | | | Jaundice | | Asterixis | | Encephalopathy or coma | | | Death |
| REMARK: Jaundice is not an AE, but occurs when the liver is not working properly or when a bile duct is blocked. It is graded as a result of liver dysfunction/failure or elevated bilirubin.  ALso CONSIDER: Bilirubin (hyperbilirubinemia). | | | | | | | | | | | | | |
| Pancreatitis | | Pancreatitis | Asymptomatic, enzyme elevation and/or radiographic findings | | | Symptomatic, medical intervention indicated | | Interventional radiology or operative intervention indicated | | Life-threatening consequences (e.g., circulatory failure, hemorrhage, sepsis) | | | Death |
| ALSO CONSIDER: Amylase | | |
| Hepatobiliary /Pancreas –Other (Specify, ) | | Hepatobiliary – Other (Specify) | Mild | | | Moderate | | Severe | | Life-threatening; disabling | | | Death |
| **INFECTION** | | | | | | | | | | | | | |
| Colitis, infectious (e.g., Clostridium difficile) | Colitis, infectious | | | Asymptomatic, pathologic or radiographic findings only | Abdominal pain with mucus and/or blood in stool | | IV antibiotics or TPN indicated | | Life-threatening consequences (e.g., perforation, bleeding, ischemia, necrosis or toxic megacolon); operative resection or diversion indicated | | | Death | |
| ALSO CONSIDER: Hemorrhage, GI – *Select;* Typhlitis (cecal inflammation). | | | | | | | | | | | | | |
| Febrile neutropenia (fever of unknown origin without clinically or microbiologically documented infection) (ANC <1.0 x 10^9^/L, fever ≥38.5°C) | Febrile neutropenia | | | — | — | | Present | | Life-threatening consequences (e.g., septic shock, hypotension, acidosis, necrosis) | | | Death | |
| ALSO CONSIDER: Neutrophils/granulocytes (ANC/AGC). | | | | | | | | | | | | | |
| Infection (documented clinically or microbiologically) with Grade 3 or 4 neutrophils (ANC <1.0 x 10**^9^**/L) – *Select* | Infection (documented clinically) – *Select* | | | *—* | Localized, local intervention indicated | | IV antibiotic, antifungal, or antiviral intervention indicated; interventional radiology or operative intervention indicated | | Life-threatening consequences (e.g., septic shock, hypotension, acidosis, necrosis) | | | Death | |
| REMARK: Fever with Grade 3 or 4 neutrophils in the absence of documented infection is graded as Febrile neutropenia (fever of unknown origin without clinically or microbiologically documented infection).  ALSO CONSIDER: Neutrophils/granulocytes (ANC/AGC). | | | | | | | | | | | | | |
| Infection with normal ANC or Grade 1 or 2 neutrophils – Select | Infection with normal ANC – *Select* | | | *—* | Localized, local intervention indicated | | IV antibiotic, antifungal, or antiviral intervention indicated; interventional radiology or operative intervention indicated | | Life-threatening consequences (e.g., septic shock, hypotension, acidosis, necrosis) | | | Death | |
| Infection with unknown ANC – *Select* Se/ect'AEs appear at the end of the CATEGORY. | Infection with unknown ANC – *Select* | | | *—* | Localized, local intervention indicated | | IV antibiotic, antifungal, or antiviral intervention indicated; interventional radiology or operative intervention indicated | | Life-threatening consequences (e.g., septic shock, hypotension, acidosis, necrosis) | | | Death | |
| REMARK: Infection with unknown ANC – *Select* is to be used in the rare case when ANC is unknown. | | | | | | | | | | | | | |
| Opportunistic infection associated with ≥Grade 2 Lymphopenia | Opportunistic infection | | | — | Localized, local intervention indicated | | IV antibiotic, antifungal, or antiviral intervention indicated; interventional radiology or operative intervention indicated | | Life-threatening consequences (e.g., septic shock, hypotension, acidosis, necrosis) | | | Death | |
| ALSO CONSIDER: Lymphopenia. | | | | | | | | | | | | | |
| Viral hepatitis | Viral hepatitis | | | Present; transaminases and liver function normal | Transaminases abnormal, liver function normal | | Symptomatic Iiver dysfunction; fibrosis by biopsy; compensated cirrhosis | | Decompensated liver function (e.g., ascites, coagulopathy, encephalopathy, coma) | | | Death | |
| REMARK: Non-viral hepatitis is graded as Infection – Select. | | | | | | | | | | | | | |
| Also CONSIDER: Albumin, serum-low (hypoalbuminemia); ALT, SGPT (serum glutamic pyruvic transaminase); AST, SGOT (serum glutamic oxaloacetic transaminase); Bilirubin (hyperbilirubinemia); Encephalopathy. | | | | | | | | | | | | | |
| Infection – Other (Specify) | Infection – Other (Specify) | | | Mild | Moderate | | Severe | | Life-threatening; disabling | | | Death | |
| **LYMPHATICS** | | | | | | | | | | | | | |
| Dermal change lymphedema, phlebolymphedema | Dermal change | | | Trace thickening or faint discoloration | Marked discoloration; leathery skin texture; papillary formation | | — | | — | | | — | |
| REMARK: Dermal change lymphedema, phlebolymphedema refers to changes due to venous stasis. | | | | | | | | | | | | | |
| ALso CONSIDER: Ulceration. | | | | | | | | | | | | | |
| Edema: head and neck | Edema: head and neck | | | Localized to dependent areas, no disability or functional impairment | Localized facial or neck edema with functional impairment | | Generalized facial or neck edema with functional impairment (e.g., difficulty in turning neck or opening mouth compared to baseline) | | Severe with ulceration or cerebral edema; tracheotomy or feeding tube indicated | | | Death | |
| Edema: limb | Edema: limb | | | 5 – 10% inter-limb discrepancy in volume or circumference at point of greatest visible difference; swelling or obscuration of anatomic architecture on close inspection; pitting edema | >10 – 30% inter-limb discrepancy in volume or circumference at point of greatest visible difference; readily apparent obscuration of anatomic architecture; obliteration of skin folds; readily apparent deviation from normal anatomic contour | | >30% inter-limb discrepancy in volume; lymphorrhea; gross deviation from normal anatomic contour, interfering with ADL | | Progression to malignancy (i.e., lymphangiosarcoma); amputation indicated; disabling | | | Death | |
| Edema: trunk/genital | Edema: trunk/genital | | | Swelling or obscuration of anatomic architecture on close inspection; pitting edema | Readily apparent obscuration of anatomic architecture; obliteration of skin folds; readily apparent deviation from normal anatomic contour | | Lymphorrhea; interfering with ADL; gross deviation from normal anatomic contour | | Progression to malignancy (i.e., lymphangiosarcoma); disabling | | | Death | |
| Edema: viscera | Edema: viscera | | | Asymptomatic; clinical or radiographic findings only | Symptomatic; intervention indicated | | Symptomatic and unable  to aliment adequately orally; interventional radiology or operative intervention indicated | | Life threatening consequences | | | Death | |
| Lymphatics – Other (Specify, _) | Lymphatics – Other (Specify, _) | | | Mild | Moderate | | Severe | | Life-threatening; disabling | | | Death | |
| **METABOLIC/ LABORATORY** | | | | | | | | | | | | | |
| Alkaline phosphatase | Alkaline phosphatase | | | >ULN - 2.5 x ULN | >2.5 - 5.0 x ULN | | >5.0 - 20.0 x ULN | | >20.0 x ULN | | | - | |
| ALT, SGPT (serum glutamic pyruvic transaminase) | ALT | | | >ULN - 2.5 x ULN | >2.5 - 5.0 x ULN | | >5.0 - 20.0 x ULN | | >20.0 x ULN | | | - | |
| AST, SGOT (serum glutamic oxaloacetic transaminase) | AST | | | >ULN - 2.5 x ULN | >2.5 - 5.0 x ULN | | >5.0 - 20.0 x ULN | | >20.0 x ULN | | | - | |
| Bilirubin (hyperbilirubinemia) | Bilirubin | | | >ULN - 1.5 x ULN | >1.5 - 3.0 x ULN | | >3.0 - 10.0 x ULN | | >10.0 x ULN | | | - | |
| REMARK: Jaundice is not an AE, but may be a manifestation of liver dysfunction/failure or elevated bilirubin. If jaundice is associated with elevated bilirubin, grade bilirubin. | | | | | | | | | | | | | |
| CPK (creatine phosphokinase) | CPK | | | >ULN - 2.5 x ULN | >2.5 x ULN - 5 x ULN | | >5 x ULN - 10 x ULN | | >10 x ULN | | | Death | |
| Creatinine | Creatinine | | | >ULN - 1.5 x ULN | >1.5 - 3.0 x ULN | | >3.0 - 6.0 x ULN | | >6.0 x ULN | | | Death | |
| ALso CONSIDER: Glomerular filtration rate. | | | | | | | | | | | | | |
| Phosphate, serum-low (hypophosphatemia) | Hypophosphatemia | | | <LLN - 2.5 mg/dL <LLN - 0.8 mmol/L | <2.5 - 2.0 mg/dL <0.8 - 0.6 mmol/L | | <2.0 - 1.0 mg/dL <0.6 - 0.3 mmol/L | | <1.0 mg/dL <0.3 mmol/L | | | Death | |
| Potassium, serum-high (hyperkalemia) | Hyperkalemia | | | >ULN - 5.5 mmol/L | >5.5 - 6.0 mmol/L | | >6.0 - 7.0 mmol/L | | >7.0 mmol/L | | | Death | |
| Potassium, serum-Iow (hypokalemia) | Hypokalemia | | | <LLN – 3.0 mmol/L | — | | <3.0 – 2.5 mmol/L | | <2.5 mmol/L | | | Death | |
| Sodium, serum-Iow (hyponatremia) | Hyponatremia | | | <LLN – 130 mmol/L | — | | <130 – 120 mmol/L | | <120 mmol/L | | | Death | |
| Uric acid, serum-high (hyperuricemia) | Hyperuricemia | | | >ULN – 10 mg/dL ≤0.59 mmol/L without physiologic consequences | — | | >ULN – 10 mg/dL ≤0.59 mmol/L with physiologic consequences | | >10 mg/dL >0.59 mmol/L | | | Death | |
| ALSO CONSIDER: Creatinine; Potassium, serum-high (hyperkalemia); Renal failure; Tumor lysis syndrome. | | | | | | | | | | | | | |
| Metabolic/Laboratory –Other (Specify) | Metabolic/Lab – Other (Specify) | | | Mild | Moderate | | Severe | | Life-threatening; disabling | | | Death | |
| **MUSCULOSKELETAL/SOFT TISSUE** | | | | | | | | | | | | | |
| Joint-effusion | Joint-effusion | | | Asymptomatic, clinical or radiographic findings only | Symptomatic; interfering with function but not interfering with ADL | | Symptomatic and interfering with ADL | | Disabling | | | Death | |
| Also CONSIDER: Arthritis (non-septic). | | | | | | | | | | | | | |
| Joint-function | Joint-function | | | Stiffness interfering with athletic activity; ≤25% Ioss of range of motion (ROM) | Stiffness interfering with function but not interfering with ADL; >25 – 50% decrease in ROM | | Stiffness interfering with ADL; >50 – 75% decrease in ROM | | Fixed or non-functional joint (arthrodesis); >75% decrease in ROM | | | — | |
| Also CONSIDER: Arthritis (non-septic). | | | | | | | | | | | | | |
| Musculoskeletal/ SoftTissue — Other (Specify) | Musculoskeletal — Other (Specify) | | | Mild | Moderate | | Severe | | Life-threatening; disabling | | Death | | |
| **NEUROLOGY** | | | | | | | | | | | | | |
| Cognitive disturbance | Cognitive disturbance | | | Mild cognitive disability; not interfering with work/school/life performance; specialized educational services/devices not indicated | Moderate cognitive disability; interfering with work/school/life performance but capable of independent living; specialized resources on part-time basis indicated | | Severe cognitive disability; significant impairment of work/school/Iife performance | | Unable to perform ADL; full-time specialized resources or institutionalization indicated | | Death | | |
| REMARK: Cognitive disturbance may be used for Attention Deficit Disorder (ADD). | | | | | | | | | | | | | |
| Confusion | Confusion | | | Transient confusion, disorientation, or attention deficit | Confusion, disorientation, or attention deficit interfering with function, but not interfering with ADL | | Confusion or delirium interfering with ADL | | Harmful to others or self; hospitalization indicated | | Death | | |
| REMARK: Attention Deficit Disorder (ADD) is graded as Cognitive disturbance. | | | | | | | | | | | | | |
| Dizziness | Dizziness | | | With head movements or nystagmus only; not interfering with function | Interfering with function, but not interfering with ADL | | Interfering with ADL | | Disabling | | Death | | |
| REMARK: Dizziness includes disequilibrium, Iightheadedness, and vertigo. | | | | | | | | | | | | | |
| ALSO CONSIDER: Neuropathy: cranial – Select Syncope (fainting). | | | | | | | | | | | | | |
| Memory impairment | Memory impairment | | | Memory impairment not interfering with function | Memory impairment interfering with function, but not interfering with ADL | | Memory impairment interfering with ADL | | Amnesia | | — | | |
| Mood alteration – *Select –* Agitation – Anxiety – Depression – Euphoria | Mood alteration – *Select* | | | Mild mood alteration not interfering with function | Moderate mood alteration interfering with function, but not interfering with ADL; medication indicated | | Severe mood alteration interfering with ADL | | Suicidal ideation; danger to self or others | | Death | | |
| Neuropathy: cranial – Select | Neuropathy: cranial – *Select* | | | Asymptomatic, detected on exam/testing only | Symptomatic, not interfering with ADL | | Symptomatic, interfering with ADL | | Life-threatening; disabling | | Death | | |
| – CN I Smell – CN II Vision – CN III Pupil, upper eyelid, extra ocular movements – CN IV Downward, inward movement of eye – CN V Motor- jaw muscles; Sensory-facial – CN VI Lateral deviation of eye – CN VII Motor-face; Sensory-taste –CN VIII Hearing and balance – CN IX Motor-pharynx; Sensory-ear, pharynx, tongue – CN X Motor-palate; pharynx, larynx – CN XI Motor-stemomastoid and trapezius – CN XII Motor-tongue | | | | | | | | | | | | | |
| Neuropathy: sensory | Neuropathy-sensory | | | Asymptomatic; loss of deep tendon reflexes or paresthesia (including tingling) but not interfering with function | Sensory alteration or paresthesia (including tingling), interfering with function, but not interfering with ADL | | Sensory alteration or paresthesia interfering with ADL | | Disabling | | Death | | |
| REMARK: Cranial nerve sensory neuropathy is graded as Neuropathy: cranial – *Select.* | | | | | | | | | | | | | |
| Seizure | Seizure | | | — | One brief generalized seizure; seizure(s) wellcontrolled by anticonvulsants or infrequent focal motor seizures not interfering with ADL | | Seizures in which consciousness is altered; poorly controlled seizure disorder, with breakthrough generalized seizures despite medical intervention | | Seizures of any kind which are prolonged, repetitive, or difficult to control (e.g., status epilepticus, intractable epilepsy) | | Death | | |
| Somnolence/ depressed level of consciousness | Somnolence | | | — | Somnolence or sedation interfering with function, but-not interfering-with ADL | | Obtundation or stupor; difficult to arouse; interfering with- ADL | | Coma | | Death | | |
| Syncope (fainting) | Syncope (fainting) | | | — | — | | Present | | Life-threatening consequences | | Death | | |
| ALSO CONSIDER: CNS cerebrovascular ischemia; Conduction abnormality/atrioventricular heart block - *Select;* Dizziness; Supraventricular and nodal arrhythmia - *Select,* Vasovagal episode; Ventricular arrhythmia - *Select.* | | | | | | | | | | | | | |
| NAVIGATION NOTE: Taste alteration (CN VII, IX) is graded as Taste alteration (dysgeusia) in the GASTROINTESTINAL CATEGORY. | | | | | | | | | | | | | |
| Neurology - Other (Specify, ) | Neurology - Other (Specify) | | | Mild | Moderate | | Severe | | Life-threatening; disabling | | Death | | |
| **OCULAR/VISUAL** | | | | | | | | | | | | | |
| Dry eye syndrome | Dry eye | | | Mild, intervention not indicated | Symptomatic, interfering with function but not interfering with ADL; medical intervention indicated | | Symptomatic or decrease in visual acuity interfering with ADL; operative intervention indicated | | — | | — | | |
| Ocular surface disease | Ocular surface disease | | | Asymptomatic or minimally symptomatic but not interfering with function | Symptomatic, interfering with function but not interfering with ADL, topical antibiotics or other topical intervention indicated | |  | | — | | — | | |
| REMARK: Ocular surface disease indudes conjunctivitis, keratoconjunctivitis sicca, chemosis, keratinization, and palpebral conjunctival epithelial metaplasia. | | | | | | | | | | | | | |
| Optic disc edema | Optic disc edema | | | Asymptomatic | Decreased visual acuity (20/40 or better); visual field defect present | | Decreased visual acuity (worse than 20/40); marked visual field defect but sparing the central 20 degrees | | Blindness (20/200 or worse) | | — | | |
| ALSO CONSIDER: Neuropathy: cranial – *Select.* | | | | | | | | | | | | | |
| Vision-blurred vision | Blurred vision | | | Symptomatic not interfering with function | Symptomatic and interfering with function, but not interfering with ADL | | Symptomatic and interfering with ADL | | Disabling | | — | | |
| Vitreous hemorrhage | Vitreous hemorrhage | | | Asymptomatic, clinical findings only | Symptomatic, interfering with function, but not interfering with ADL; intervention not indicated | | Symptomatic, interfering with ADL; vitrectomy indicated | | — | | — | | |
| Watery eye (epiphora, tearing) | Watery eye | | | Symptomatic, intervention not indicated | Symptomatic, interfering with function but not interfering with ADL | | Symptomatic, interfering with ADL | | — | | — | | |
| Ocular/Visual – Other (Specify) | Ocular – Other (Specify) | | | Symptomatic not interfering with function | Symptomatic and interfering with function, but not interfering with ADL | | Symptomatic and interfering with ADL | | Blindness (20/200 or worse) | | Death | | |
| **PAIN** | | | | | | | | | | | | | |
| Pain | Pain | | | Mild pain not interfering with function | Moderate pain; pain or analgesics interfering with function, but not interfering with ADL | | Severe pain; pain or analgesics severely interfering with ADL | | Disabling | | — | | |
| **PULMONARY/UPPER RESPIRATORY** | | | | | | | | | | | | | |
| Cough | Cough | | | Symptomatic, non- narcotic medication only indicated | Symptomatic and narcotic medication indicated | | Symptomatic and significantly interfering with sleep or ADL | | — | | — | | |
| Dyspnea (shortness of breath) | Dyspnea | | | Dyspnea on exertion, but can walk 1 flight of stairs without stopping | Dyspnea on exertion but unable to walk 1 flight of stairs or 1 city block (0.1 km) without stopping | | Dyspnea with ADL | | Dyspnea at rest; intubation/ ventilator indicated | | Death | | |
| Also CONSIDER: Hypoxia; Neuropathy: motor; Pneumonitis/pulmonary infiltrates; Pulmonary fibrosis (radiographic changes). | | | | | | | | | | | | | |
| Pleural effusion  (non-malignant) | Pleural effusion | | | Asymptomatic | Symptomatic,  intervention such as diuretics or up to 2 therapeutic thoracenteses indicated | | Symptomatic and  supplemental oxygen, >2 therapeutic thoracenteses, tube drainage, or pleurodesis indicated | | Life-threatening (e.g., causing hemodynamic instability or ventilatory support indicated) | | Death | | |
| Also CONSIDER: Atelectasis; Cough; Dyspnea (shortness of breath); Hypoxia; Pneumonitis/pulmonary infiltrates; Pulmonary fibrosis (radiographic changes). | | | | | | | | | | | | | |
| Pneumonitis/ pulmonary infiltrates | Pneumonitis | | | Asymptomatic,  radiographic findings only | Symptomatic, not interfering with ADL | | Symptomatic, interfering with ADL; 0_2_ indicated | | Life-threatening;  ventilatory support indicated | | Death | | |
| Also CONSIDER: Adult Respiratory Distress Syndrome (ARDS); Cough; Dyspnea (shortness of breath); Hypoxia; Infection (documented clinically or microbiologically) with Grade 3 or 4  neutrophils (ANC <1.0 x 10^9^/L) - *Select;* Infection with normal ANC or Grade 1 or 2 neutrophils - *Select;* Infection with unknown ANC - *Select;* Pneumonitis/pulmonary infiltrates; Pulmonary fibrosis (radiographic changes). | | | | | | | | | | | | | |
| Pulmonary fibrosis (radiographic changes) | Pulmonary fibrosis | | | Minimal radiographic findings (or patchy or bi-basilar changes) with estimated radiographic proportion of total lung volume that is fibrotic of <25% | Patchy or bi-basilar changes with estimated radiographic proportion of total lung volume that is fibrotic of 25 – <50% | | Dense or widespread infiltrates /consolidation with estimated radiographic proportion of total lung volume that is fibrotic of 50 – <75% | | Estimated radiographic proportion of total lung volume that is fibrotic is ≥75%; honeycombing | | Death | | |
| REMARK: Fibrosis is usually a "late effect" seen >3 months after radiation or combined modality therapy (including surgery). It is thought to represent scar/fibrotic lung tissue. It may be dfficult to distinguish from pneumonitis that is generally seen within 3 months of radiation or combined modality therapy. ALso CONSIDER: Adult Respiratory Distress Syndrome (ARDS); Cough; Dyspnea (shortness of breath); Hypoxia; Infection (documented clinically or microbiologically) with Grade 3 or 4 neutrophils (ANC <1.0 x 10^9^/L) – Select; Infection with normal ANC or Grade 1 or 2 neutrophils – Select; Infection with unknown ANC – Select. | | | | | | | | | | | | | |
| Pulmonary/Upper Respiratory – Other (Specify) | Pulmonary – Other (Specify) | | | Mild | Moderate | | Severe | | Life-threatening; disabling | | Death | | |
| **RENAL/GENITOURINARY** | | | | | | | | | | | | | |
| Renal failure | Renal failure | | | — | — | | Chronic dialysis not indicated | | Chronic dialysis or renal transplant indicated | | Death | | |
| ALSO CONSIDER: Glomerular filtration rate. | | | | | | | | | | | | | |
| Urinary frequency/urgency | Urinary frequency | | | Increase in frequency or nocturia up to 2 x normal; enuresis | Increase >2 x normal but <hourly | | ≥1 x/hr; urgency; catheter indicated | | — | | — | | |
| Renal/ Genitourinary – Other (Specify, ) | Renal – Other (Specify) | | | Mild | Moderate | | Severe | | Life-threatening; disabling | | Death | | |
| **SEXUAL/REPRODUCTIVE FUNCTION** | | | | | | | | | | | | | |
| Breast volume/hypoplasia | Breast | | | Minimal asymmetry; minimal hypoplasia | Asymmetry exists, ≤1/3 of the breast volume; moderate hypoplasia | | Asymmetry exists, >1/3 of the breast volume; severe hypoplasia | | — | | — | | |
| REMARK: Breast volume is referenced with both arms straight overhead. | | | | | | | | | | | | | |
| Erectile dysfunction | Erectile dysfunction | | | Decrease in erectile function (frequency/rigidity of erections) but erectile aids not indicated | Decrease in erectile function (frequency/rigidity of erections), erectile aids indicated | | Decrease in erectile function (frequency/rigidity of erections) but erectile aids not helpful; penile prosthesis indicated | | — | | — | | |
| Ejaculatory dysfunction | Ejaculatory dysfunction | | | Diminished ejaculation | Anejaculation or retrograde ejaculation | | — | | — | | — | | |
| Gynecomastia | Gynecomastia | | | — | Asymptomatic breast enlargement | | Symptomatic breast enlargement; intervention indicated | | — | | — | | |
| ALSO CONSIDER: Pain – *Select.* | | | | | | | | | | | | | |
| Infertility/sterility | Infertility/sterility | | | — | Male: oligospermial/low sperm count Female: diminished fertility/ ovulation | | Male: sterile/azoospermia Female: infertile/ anovulatory | | — | | — | | |
| Irregular menses (change from baseline) | Irregular menses | | | 1 – 3 months without menses | >3 – 6 months without menses but continuing menstrual cycles | | Persistent amenorrhea for >6 months | | — | | — | | |
| Libido | Libido | | | Decrease in interest but not affecting relationship; intervention not indicated | Decrease in interest and adversely affecting relationship; intervention indicated | | — | | — | | — | | |
| Orgasmic dysfunction | Orgasmic function | | | Transient decrease | Decrease in orgasmic response requiring intervention | | Complete inability of orgasmic response; not responding to intervention | | — | | — | | |
| Sexual/Reproductive Function – Other (Specify, ) | Sexual – Other (Specify) | | | Mild | Moderate | | Severe | | Disabling | | Death | | |
| **VASCULAR** | | | | | | | | | | | | | |
| Phlebitis (including  superficial thrombosis) | Phlebitis | | | — | Present | |  | |  | | Death | | |
| Thrombosis/ thrombus/ embolism | Thrombosis/  thrombus/ embolism | | | — | Deep vein thrombosis or cardiac thrombosis; intervention (e.g., anticoagulation, lysis, filter, invasive procedure) not indicated | | Deep vein thrombosis or cardiac thrombosis; intervention (e.g., anticoagulation, lysis, filter, invasive procedure) indicated | | Embolic event including Pulmonary embolism or life threatening thrombus | | Death | | |
| Vascular – Other (Specify, ) | Vascular – Other (Specify) | | | Mild | Moderate | | Severe | | Life-threatening; disabling | | Death | | |

ADL : Activities of Daily Living

ANC : Absolute Neutrophil Count

AGC : Absolute Granulocyte Count

BSA : Body Surface Area

CHF : Congestive Heart Failure

GVHD : Graft-versus-Host-Disease

ICP : Intracranial Pressure

TPN : Total Parenteral Nutrition

## Appendix 11 : IIEF-5 questionnaire

| **In the Last 3 weeks...**  **How do you rate your confidence that you could get and keep an erection..?**  Very Low      Low      Moderate      High      Very High |
| --- |
| **When you had erections with sexual stimulation, how often were your erections hard enough for penetration..?**  Never/almost never   A few times   Sometimes (50:50)  Most times  Almost always/always |
| **During sexual intercourse, how often were you able to maintain your erection after you had penetrated (entered) your partner..?**  Never/almost never   A few times   Sometimes (50:50)  Most times  Almost always/always |
| **During sexual intercourse, how difficult was it to maintain your erection to completion of intercourse..?**  Extremely difficult    Very difficult    Difficult    Slightly difficult    Not difficult |
| **When you attempted sexual intercourse, how often was it satisfactory for you..?**  Never/almost never   A few times   Sometimes (50:50)  Most times  Almost always/always |

## Appendix 12 : The International Prostate Symptom Score (IPSS)

| **Please answer the following questions about your urinary symptoms. Write your score for each question at the end of each row.** | | | | | | | |
| --- | --- | --- | --- | --- | --- | --- | --- |
| **Over the past month, how often have you...** | **Not at all** | **Less than 1 time in 5** | **Less than half the time** | **About half the time** | **More than half the time** | **Almost always** | **Your Score** |
| **1.** ...had a sensation of not emptying your bladder completely after you finished urinating? | 0 | 1 | 2 | 3 | 4 | 5 |  |
| **2.** ...had to urinate again less than two hours after you finished urinating? | 0 | 1 | 2 | 3 | 4 | 5 |  |
| **3.** ...stopped and started again several times when you urinated? | 0 | 1 | 2 | 3 | 4 | 5 |  |
| **4.** ...found it difficult to postpone urination? | 0 | 1 | 2 | 3 | 4 | 5 |  |
| **5.** ...had a weak urinary stream? | 0 | 1 | 2 | 3 | 4 | 5 |  |
| **6.** ...had to push or strain to begin urination? | 0 | 1 | 2 | 3 | 4 | 5 |  |
|  | | | | | | | |
| **And finally..** | **None** | **Once** | **Twice** | **3 times** | **4 times** | **5 times or more** |  |
| **7.** Over the past month, how many times did you most typically get up to urinate from the time you went to bed at night until the time you got up in the morning? | 0 | 1 | 2 | 3 | 4 | 5 |  |
| **Add up your total score and write it in the box.** | | | | | | | **Total** |

**Supplementary question - Quality of life due to urinary symptoms.**

If you were to spend the rest of your life with your urinary condition the way it is now, how would you feel about that? (Please tick which best describes how you would feel.)

0. Delighted
1. Pleased
2. Mostly satisfied
3. Mixed - about equally satisfied and dissatisfied
4. Mostly dissatisfied
5. Unhappy
6. Terrible
